# The Prognostic Value of the *XPC rs2228001* Single Nucleotide Polymorphism in Cholangiocarcinoma

**DOI:** 10.1111/liv.70292

**Published:** 2025-08-20

**Authors:** Guanwu Wang, Anna Mantas, Dong Liu, Tarick M. Al‐Masri, Smiths Sengkwawoh Lueong, Jens Siveke, Tom Luedde, Tom F. Ulmer, Iakovos Amygdalos, Florian W. R. Vondran, Georg Lurje, Ulf Peter Neumann, Lara R. Heij, Jan Bednarsch

**Affiliations:** ^1^ Department of Surgery and Transplantation University Hospital RWTH Aachen Aachen Germany; ^2^ Department of Hepatobiliary Surgery Hunan Provincial People's Hospital (The First Affiliated Hospital of Hunan Normal University) Changsha China; ^3^ Department of Surgery and Transplantation University Hospital Essen Essen Germany; ^4^ University of Applied Science Aachen Aachen Germany; ^5^ German Cancer Consortium (DKTK), Partner Site Essen, a Partnership Between German Cancer Research Center (DKFZ) and University Hospital Essen Essen Germany; ^6^ Bridge Institute of Experimental Tumor Therapy (BIT) and Division of Solid Tumor Translational Oncology (DKTK), west German Cancer Center, University Hospital Essen, University of Duisburg‐Essen Essen Germany; ^7^ Department of Gastroenterology, Hepatology and Infectious Diseases Heinrich Heine University Duesseldorf Duesseldorf Germany; ^8^ Department of Surgery Maastricht University Medical Centre (MUMC) Maastricht the Netherlands; ^9^ Department of Surgery and Transplantation University Hospital Heidelberg Heidelberg Germany; ^10^ Institute of Pathology University Hospital Essen Essen Germany

**Keywords:** Cholangiocellular carcinoma, DNA repair genes, oncological outcome, single nucleotide polymorphisms, *XPC rs2228001*

## Abstract

**Background and Aims:**

Cholangiocarcinoma (CCA) is one of the most prevalent primary liver malignancies with increasing incidence and mortality rates, particularly in Southeast Asia. Surgical resection is a primary therapeutic option offered to patients with localised disease. Unfortunately, early disease recurrence is common and robust tools for tailored patient‐centric post‐operative management and follow‐up schemes are highly desired but currently lacking. To address this unmet clinical need, this study investigated the clinical utility of the single nucleotide polymorphisms (SNPs) in DNA repair genes (*ERCC5 rs1047768, APEX1 rs1130409, PARP1 rs1805414, XPC rs2228001* and *ERCC5 rs873601*) and patient prognosis after curative intent surgery.

**Methods:**

A cohort of 229 patients who underwent surgical treatment for intrahepatic (iCCA) and perihilar (pCCA) cholangiocarcinoma was examined. Kaplan–Meier and multivariable Cox regression analyses were used to assess the impact of these SNPs on recurrence‐free survival (RFS), cancer‐specific survival (CSS) and overall survival (OS).

**Results:**

Within comprehensive multivariable analyses, the TT genotype of *XPC rs2228001* was significantly associated with prolonged RFS (GG/GT = 1, HR = 0.50, *p* = 0.027), CSS (HR = 0.42, *p* = 0.018) and OS (HR = 0.31; *p* = 0.031) in iCCA. Similarly, in pCCA, the TT genotype *of XPC rs2228001* was an independent prognostic factor for prolonged CSS (GG/GT = 1, HR = 0.48, *p* = 0.041).

**Conclusions:**

These findings suggest that SNPs in DNA repair genes, particularly *XPC rs2228001*, play a crucial role in modulating the prognosis of CCA.

**Summary**

- This study found that a genetic variation known as *XPC rs2228001* can predict the survival of patients with cholangiocarcinoma (a type of liver cancer) after surgery.
- Patients carrying a specific form of this genetic variant (the TT genotype) had significantly better outcomes, with longer periods before cancer recurrence and improved overall survival.
- Identifying this genetic marker may help doctors better manage and monitor patients following surgery.

**Abbreviations**
AD: allelic discrimination
APEX1: apurinic/apyrimidinic endodeoxyribonuclease 1
ASA: American Society of anesthesiologists
BER: base excision repair
BMI: Body Mass Index
CCA: cholangiocarcinoma
CSS: cancer‐specific survival
dCCA: distal cholangiocarcinoma
DDR: DNA damage repair
FFP: Fresh frozen plasma
HR: Hazard ratio
HWE: Hardy–Weinberg equilibrium
iCCA: intrahepatic cholangiocarcinoma
ICU: intensive care unit
LVI: lymph vascular invasion
MVI: microvascular invasion
NER: nucleotide excision repair
NHEJ: non‐homologous end joining
OGG1: 8‐oxoguanine glycosylase
OS: overall survival
PARP‐1: poly (ADP‐Ribose) polymerase 1
pCCA: perihilar cholangiocarcinoma
PRBC: packed red blood cells
RFS: recurrence‐free survival
SNPs: single nucleotide polymorphisms
XPC: xeroderma pigmentosum complementation group C

## 1 Background

Cholangiocarcinoma (CCA) accounts for 10%–20% of all primary liver cancer cases and approximately 3% of all gastrointestinal malignancies [1]. CCA is histologically classified into three major subclasses including: intrahepatic (iCCA), perihilar (pCCA) and distal (dCCA) subtypes [2]. Although incidence and corresponding mortality rates are relatively low in most western countries (0.3–6 per 100 000 individuals annually), CCA is a major public health concern with a focus on Southeast Asia (7.1–14.5 per 100 000 individuals annually) [3]. Higher incidence rates are observed among older males owing to several risk factors such as obesity, viral hepatitis, primary sclerosing cholangitis, diabetes mellitus, hepatolithiasis and liver fluke infection [4]. Radical surgical excision remains the mainstay curative intent treatment modality but is applicable in only about 25% of cases, often due to late stages at disease diagnosis. Despite multimodal treatments, post‐operative recurrence rates are still high (50%–70%) and the overall 5‐year survival rate is still unacceptably below 40% [5, 6, 7]. Tailoring patient management schemes to recurrence risk can improve outcome and patient life quality. Unfortunately, however, tools for such patient‐centric management schemes are still missing.

A single nucleotide polymorphism (SNP) is a DNA sequence variation occurring when a single nucleotide differs among individuals, typically found about once in every 1000 nucleotides throughout the human genome [8]. SNPs can either be inherited or occur as a result of assaults to the genetic material including insertions, deletions, or rearrangements. These genetic alterations, in some instances, might not have deleterious effects on the resulting protein sequence (synonymous alterations) and therefore only contribute to genetic diversity. Unfortunately, some of these alterations alter both the protein sequence and function (non‐synonymous alteration), leading to disease conditions under certain circumstances [9]. SNPs have been reported to be associated with some clinical disease phenotypes and therefore can serve as genetic markers, helping to explore the correlation between genotype and disease susceptibility, overall patient outcome or drug responses [10, 11, 12, 13]. Alteration in the protein sequence of key regulatory and repair proteins, such as those involved in DNA damage repair, could alter or impair their physiological functions, resulting in increased mutation rates, neoantigens, new protein variants and genomic instability, contributing to tumorigenesis [14]. Notably, SNPs in DNA repair genes may influence patient outcomes after surgical resection, as impaired DNA repair can lead to increased mutation rates, tumour progression and recurrence, all of which can negatively impact survival rates [15, 16, 17, 18].

Thus, this study aims to investigate the relationship between single nucleotide polymorphisms (SNPs) in DNA repair genes (*ERCC5 rs1047768 and rs873601, APEX1 rs1130409, PARP1 rs1805414* and *XPC rs2228001*) and the prognosis of CCA. These SNPs were selected based on their established biological relevance in DNA repair pathways and previous associations with clinical outcomes in various malignancies [15].

## 2 Methods

### 2.1 Study Population

Patients enrolled in this study underwent curative intent surgical resection for iCCA and pCCA at the RWTH Aachen University Hospital between May 2009 and December 2020. A total of 229 patients with no post‐operative mortality were included, comprising 117 patients with pCCA and 112 patients with iCCA. The study was approved by the Institutional Review Board of RWTH Aachen University, adhering to Good Clinical Practice guidelines and the current Declaration of Helsinki.

### 2.2 DNA Extraction and Quantification

Nontumor tissue specimens were procured from formalin‐fixed, paraffin‐embedded (FFPE) blocks of liver tissue samples of 229 patients with CCA, encompassing 117 pCCA and 112 iCCA cases, archived at the RWTH Aachen University Hospital. Haematoxylin‐ and eosin‐stained slides of all specimens were examined by a single pathologist. Genomic DNA was extracted from FFPE material utilising the QIAamp DNA FFPE Tissue Kit (Qiagen, Hilden, Germany). Briefly, 10 μm sections were deparaffinised with 1 mL of xylene with vortexing for 2 min at room temperature followed by ethanol for xylene removal. Subsequently, the FFPE material was incubated with 20 μL of proteinase K and 180 μL of ATL lysis buffer at 56°C on a heating block for 1 h, followed by incubation at 90°C for 1 h. The lysate was loaded onto a DNA purification column. Following binding of the DNA to the membrane, columns were washed twice, dried and the DNA was eluted in 50 μL of elution buffer. The resulting DNA was next quantified using a BioTek spectrophotometer (Synergy HT, USA). All samples were stored at −20°C until the time of analysis and the sample concentrations were adjusted to 10 ng/μL prior to analysis. We assessed the concentration and purity of genomic DNA extracted from FFPE samples using a NanoDrop spectrophotometer (Thermo Fisher Scientific, USA). Only samples with an A260/280 ratio between 1.8 and 2.0 and a concentration ≥ 10 ng/μL were included in the subsequent genotyping analysis.

### 2.3 SNP Selection

We meticulously assessed SNPs influencing DNA repair pathways. Our selection criteria for genes analysed in this study were as follows: (a) genes must display well‐characterised polymorphisms with anticipated biological significance; (b) polymorphisms should demonstrate sufficient prevalence to facilitate robust statistical associations with clinical outcomes; (c) the relationship between the SNPs and CCA remains unexplored in the existing literature. Following these criteria, a compendium of five SNPs located on 4 gene loci was selected. In the case of a multiallelic SNP with several rare alternative alleles, only the common and clinically significant alleles were genotyped. Details are displayed in Table S1.

### 2.4 SNP Analysis

TaqMan assays (Thermo Fisher Scientific, CA, USA) were employed for SNP genotyping using the ABI 7500 Real‐Time PCR System. Assay components were supplied at a 40× concentration. Each reaction comprised a final volume of 5.05 μL, consisting of 2.5 μL of TaqMan Genotyping Master Mix, 0.25 μL of TaqMan Probe Mix, 1 μL of nuclease‐free water and 1.3 μL of genomic DNA. The PCR protocol initiated with an activation step at 95°C for 10 min, followed by 40 cycles for amplification of genomic DNA, with each cycle consisting of 15 s at 92°C and 60 s at 60°C for DNA extracted from tissue samples. To validate the results of the real‐time PCR, approximately 5% of the assays were repeated. Additionally, 10% of both positive and negative samples were randomly selected for retesting to validate the real‐time PCR outcomes. The real‐time PCR instrument's software presents allelic discrimination (AD) data graphically as Allele 1 (VIC dye) vs. Allele 2 (FAM dye). Each sample is depicted as an individual point on the AD plot. A typical AD plot displays clusters of homozygotes, a heterozygote cluster, and no‐template controls. Points within each cluster are closely grouped, with distinct separation from other clusters.

### 2.5 Statistical Analysis

Each of the 5 SNPs underwent Hardy–Weinberg equilibrium (HWE) assessment via Pearson's goodness‐of‐fit χ^2^ test, with those demonstrating equilibrium (*p* > 0.05) being included in the study. The investigation delineated patient prognoses through three salient endpoints: recurrence‐free survival (RFS), cancer‐specific survival (CSS) and overall survival (OS), each rigorously measured post‐surgery. RFS denotes the duration from surgery to the first instance of recurrence. CSS is defined as the period from surgery to the time of death due to cancer, with deaths from other causes being censored at the time of occurrence. OS is defined as from liver resection to death, without censoring perioperative mortality. Categorical variables were analysed using Fisher's exact test, and continuous variables were analysed using nonparametric tests, specifically the Mann–Whitney U test for comparisons between two independent groups and the Kruskal‐Wallis test for comparisons among more than two groups. Kaplan–Meier survival curves and log‐rank tests were employed to assess survival differences among genotypes in terms of RFS and OS. The Cox proportional hazards model facilitated a nuanced exploration of variables impacting RFS, CSS and OS, with results presented alongside 95% CIs. A *p*‐value threshold of < 0.1 was used to identify variables to be included in multivariable models to reduce type II errors and ensure relevant variables are not prematurely excluded. All statistical analyses were executed utilising SPSS Statistics (v. 26, IBM Corp, Armonk, NY, USA).

## 3 Results

### 3.1 Characteristics of the Study Cohort

The study enrolled a total of 229 CCA patients, 48.9% (112/229) of whom were treated for iCCA and 51.1% (117/227) were treated for pCCA.

**FIGURE 1:**
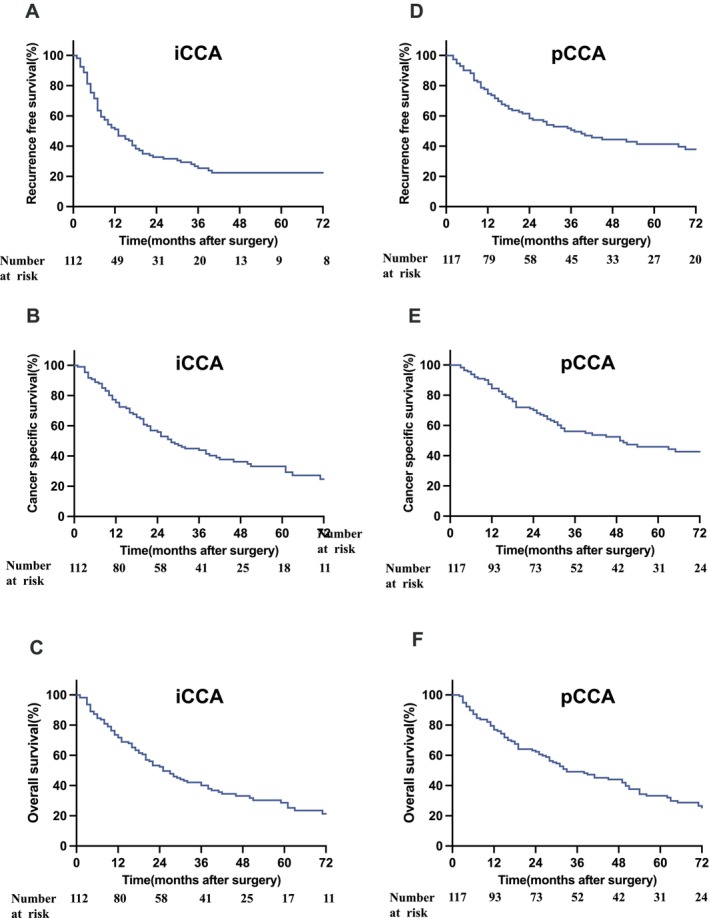
Survival data in patients with intrahepatic cholangiocarcinoma and perihilar cholangiocarcinoma. Survival data for CCA patients are presented. Among patients with iCCA, the median follow‐up period was 63 months, with a median recurrence‐free survival (RFS) of 12 months (A), cancer‐specific survival (CSS) of 28 months (B), and overall survival (OS) of 25 months (C). For patients with pCCA, the median follow‐up was 89 months, with a median RFS of 37 months (D), CSS of 49 months (E), and OS of 33 months (F). CCA, Cholangiocarcinoma; CSS, cancer‐specific survival; iCCA, intrahepatic cholangiocarcinoma; OS, overall survival; pCCA, perihilar cholangiocarcinoma; RFS, recurrence‐free survival.

Among patients with iCCA, the majority were female (56.3%) and exhibited a median age of 65 years. A significant portion of patients fell into categories II (40.2%) and III (52.7%) of the American Society of Anesthesiologists (ASA). Neoadjuvant chemotherapy was administered to only nine patients (8.0%). Most patients (91.1%) underwent major liver resections. Pathological analysis indicated an R0 rate of 77.7% and nodal metastases in 30.4% of cases. Post‐operative data highlighted shorter intensive care unit (ICU) stay, shorter hospitalisation, and lower incidences of severe complications (Clavien–Dindo IVb and V) in iCCA patients compared to pCCA patients. Following a median follow‐up of 63 months, 62.6% of patients showed disease recurrence, resulting in a median RFS of 12 months, a median CCS of 28 months, and a median OS of 25 months (Figure 1A–C).

Most patients with pCCA were male (68.6%), with a median age of 68 years. In accordance with the Bismuth classification, 6.0% of pCCA cases were classified as Bismuth I, 11.1% as Bismuth II, 24.8% as Bismuth IIIa, 28.2% as Bismuth IIIb, and 29.9% as Bismuth IV. Chemotherapy before surgery was administered in 4.3% of the cases. Notable clinical features included a high rate of pre‐operative portal vein embolization (37.9%) and pre‐operative cholangitis (25.6%). Pathologically, a high proportion of patients were categorised as pT2 stage (61.5%) with an R0 rate of 74.4% and nodal metastases observed in 42.7% of cases. The post‐operative course was characterised by prolonged ICU stays and hospitalisation as well as a higher incidence of severe complications (27.3%). After a median follow‐up of 89 months, 54.7% of patients recurred, resulting in a median RFS of 37 months, a median CCS of 49 months, and a median OS of 33 months (Figure 1D–F). Further details of both patient cohorts are summarised in Table 1.

**TABLE 1:** Patient characteristics.

Variables	iCCA (*n* = 112)	pCCA (*n* = 117)
**Demographics**		
Gender, M/F (%)	49 (43.8)/63 (56.3)	78 (66.7)39/(33.3)
Age (years)	65 (58–75)	69 (58–74)
ASA, *n* (%)		
I	3 (2.7)	6 (5.1)
II	45 (40.2)	47 (40.2)
III	59 (52.7)	58 (49.6)
IV	5 (4.5)	6 (5.1)
V	0	0
Bismuth type, *n* (%)		
I	/	7 (6.0)
II	/	13 (11.1)
IIIa	/	29 (24.8)
IIIb	/	33 (28.2)
IV	/	35 (29.9)
Cholangitis, *n* (%)	4 (3.6)	30 (25.6)
Primary sclerosing cholangitis, *n* (%)	2 (1.8)	2 (1.7)
Portal vein embolization, *n* (%)	7 (6.3)	41 (35.0)
Pre‐operative Chemotherapy, *n* (%)	9 (8.0)	5 (4.3)
**Clinical chemistry**		
AST (U/L)	34 (26–47)	47 (34–95)
ALT (U/L)	29 (20–49)	69 (36–144)
Albumin (g/dL)	4.4 (4.0–4.6)	3.9 (3.5–4.2)
AP (U/L)	118 (84–215)	254 (159–423)
CA19‐9 (U/mL)	39.5 (13.9–236.3)	85.8 (31.2–287.7)
CRP (mg/L)	7.5 (2.8–19.4)	11.8 (5.4–34.7)
GGT (U/L)	113 (65–275)	472 (216–756)
Haemoglobin (g/dL)	13.1 (12.2–14.3)	12.4 (11.2–13.3)
INR	1.0 (0.9–1.1)	1.0 (0.9–1.1)
Platelet count (/nL)	252 (202–306)	293 (229–388)
Prothrombin time (%)	100 (90–108)	96 (81–105)
Total bilirubin (mg/dL)	0.5 (0.3–0.7)	1.1 (0.5–2.8)
**Operative Data**		
Intraoperative PRBC, *n* (%)	31 (72.3)	54 (46.2)
Intraoperative FFP, *n* (%)	38 (33.9)	68 (58.1)
Operative time (minutes)	295 (230–359)	420 (356–485)
Operative procedure, *n* (%)		
Hemihepatectomy	44 (39.2)	29 (24.8)
Extended hemihepatectomy	17 (15.1)	54 (46.2)
Trisectionectomy	12 (10.8)	24 (20.6)
Hepatoduodenoectomy	0 (0)	5 (4.3)
ALPPS	10 (8.9)	1 (0.9)
Others	29 (25.8)	4 (3.4)
Time to surgery (days^a^)	45 (29–83)	38 (24–56)
**Pathological examination**		
LVI, *n* (%)	20 (17.9)	26 (22.2)
MVI, *n* (%)	5 (4.5)	2 (1.7)
R0 resection, *n* (%)	87 (77.7)	87 (74.4)
pT category *n* (%)		
1	46 (41.1)	10 (8.6)
2	46 (41.1)	72 (61.5)
3	12 (10.7)	26 (22.2)
4	8 (7.1)	9 (7.7)
pN category, *n* (%)		
N0	69 (61.6)	67 (57.3)
N1	34 (30.4)	50 (42.7)
Tumour grading, *n* (%)		
G1	0	7 (6.0)
G2	73 (65.2)	83 (70.9)
G3	26 (23.2)	22 (18.8)
G4	3 (2.7)	1 (0.9)
**Post‐operative Data**		
Intensive care, days	1 (1–1)	1 (1–2)
Hospitalisation, days	12 (8–22)	17 (12–36)
Post‐operative complications, *n* (%)		
No complications	43 (38.4)	19 (16.2)
Clavien–Dindo I	4 (3.6)	8 (6.8)
Clavien–Dindo II	24 (21.4)	33 (28.2)
Clavien–Dindo IIIa	25 (22.3)	25 (28.2)
Clavien–Dindo IIIb	10 (8.9)	19 (16.2)
Clavien–Dindo IVa	5 (4.5)	8 (6.8)
Clavien–Dindo IVb	1 (0.9)	5 (4.3)
Clavien–Dindo V	0	0
**Oncologic Data**		
Adjuvant chemotherapy, *n* (%)	42 (37.5)	31 (26.5)
Recurrence, *n* (%)	77 (68.8)	64 (54.7)
Median RFS, months (95% CI)	12 (7–17)	37 (23–51)
Median CSS, months (95% CI)	28 (21–35)	49 (24–74)
Median OS, months (95% CI)	25 (18–32)	33 (20–46)

*Note:* Data presented as median and interquartile range if not noted otherwise. Abbreviations: ALPPS, associating liver partition with portal vein ligation for staged hepatectomy; ASA, American Society of Anesthesiology; BMI, Body mass index; CSS, cancer‐specific survival; FFP, fresh frozen plasma; LVI, lymph vascular invasion; MVI, microvascular invasion; OS, Overall Survival; PRBC, packed red blood cells; RFS, recurrence free survival.

^a^ Time to surgery refers to the time frame from diagnosis to surgery.

### 3.2 SNPs In iCCA

Genotype and allele frequencies for all five SNPs analysed within this study are presented in Table S2. All SNPs conform to the HWE. Among the SNPs examined, one patient with *rs873601* (111/112) and 10 patients with *rs1047768* (102/112) failed to be genotyped.

#### 3.2.1 Kaplan–Meier Analysis

Following a median follow‐up period of 63 months, the study cohort exhibited median RFS, CCS and OS of 12, 28 and 25 months, respectively.

**FIGURE 2:**
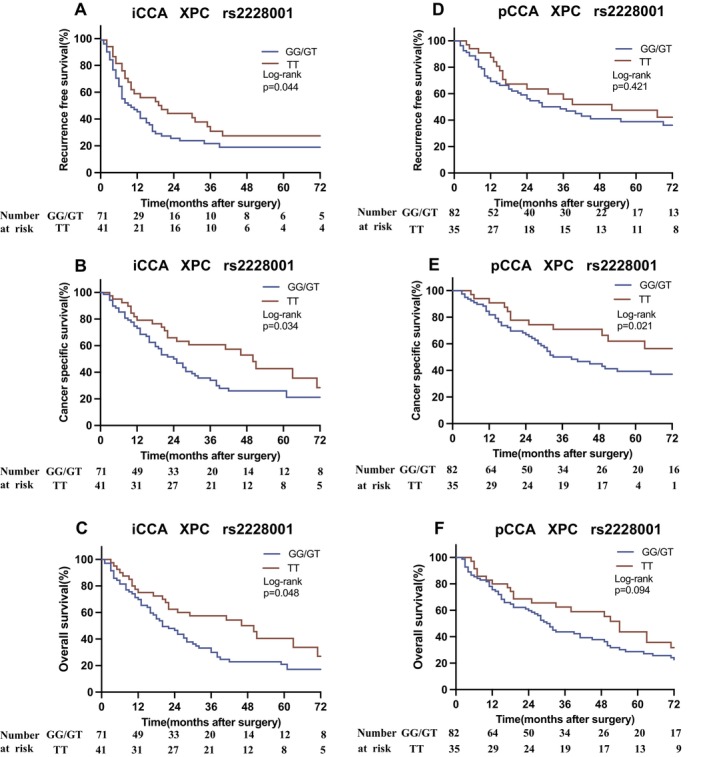
Post‐operative outcome in relation to XPC rs2228001 polymorphism in patients with cholangiocarcinoma. The impact of *XPC rs2228001* on oncological outcomes in iCCA patients is illustrated in panels (A–C). Regarding recurrence‐free survival (RFS), patients with the GG/GT genotypes had a median RFS of 9 months, compared to 20 months for those with the TT genotype (A). For cancer‐specific survival (CSS), the GG/GT genotypes were associated with a median CSS of 24 months, whereas patients with the TT genotype had a median CSS of 50 months (B). Similarly, in terms of overall survival (OS), the GG/GT genotypes exhibited a median OS of 20 months, in contrast to 46 months for the TT genotype (C).Likewise, the impact of *XPC rs2228001* on oncological outcomes in pCCA patients is depicted in panels (D–F). Regarding RFS, patients with the GG/GT genotypes had a median RFS of 82 months, compared to 35 months for those with the TT genotype (D). For CSS, the GG/GT genotypes were associated with a median CSS of 39 months, whereas patients with the TT genotype had a median CSS of 89 months (E). In terms of OS, the GG/GT genotypes demonstrated a median OS of 30 months, in contrast to 54 months for the TT genotype (F). CCA, Cholangiocarcinoma; CSS, cancer‐specific survival; iCCA, intrahepatic cholangiocarcinoma; OS, overall survival; pCCA, perihilar cholangiocarcinoma; RFS, recurrence‐free survival.

In Kaplan–Meier survival analysis, the recessive models for *APEX1 rs1130409* (*p* = 0.022) and *XPC rs2228001* (*p* = 0.044) were both statistically significant in the context of RFS. Specifically, participants with the TT/TG genotypes of *APEX1 rs1130409* were associated with a median RFS of 15 months (95% CI = 8.9–21.1), contrasting with 7 months (95% CI = 4.1–9.9) for the GG genotype (*p* = 0.024). For *XPC rs2228001*, patients with the GG/GT genotypes exhibited a median RFS of 9 months (95% CI = 5.2–12.8), compared to 20 months (95% CI = 7.6–32.4) for the TT genotype (Figure 2A). In the codominant model, a statistically significant difference was observed between the genotypes of SNP *rs2228001* (*p* = 0.015). Other SNPs were not significantly associated with RFS.

Regarding CSS, the *XPC rs2228001* SNP demonstrated statistical significance in the recessive model (*p* = 0.034). Patients with the GG/GT genotypes displayed a median CSS of 24 months (95% CI = 17.1–30.9), while those with the TT genotype exhibited a median CSS of 50 months (95% CI = 37.8–62.2) (Figure 2B). In the codominant model, a statistically significant difference was observed between the genotypes of SNP *rs2228001* (*p* = 0.019). No significant associations were observed between other SNPs and CSS.

In terms of OS, recessive models of *APEX1 rs1130409, PARP1 rs1805414* and *XPC rs2228001* all exhibited statistical significance. Regarding *APEX1 rs1130409*, the TT/TG genotypes exhibited a median OS of 27 months (95% CI = 17.8–36.2), compared to 16 months (95% CI = 2.6–29.4) for those with the GG genotype (Figure 2D). *For PARP1 rs1805414*, the AA/AG genotypes were associated with a median OS of 27 months (95% CI = 18.3–35.7), whereas the GG genotype was associated with 10 months (95% CI = 5.4–14.6). The *XPC rs2228001* GG/GT genotypes demonstrated a median OS of 20 months (95% CI = 12.9–27.1), contrasting with 46 months (95% CI = 16.7–75.3) for the TT genotype (Figure 2C). In the codominant and dominant models, genotypic variation of SNP *rs1805414* demonstrated a statistically significant difference (*p* = 0.026 and *p* = 0.033). The other SNPs did not show a significant correlation with OS. The results, including univariate Cox regressions within all models, are summarised in Table 2.

**TABLE 2:** Single nucleotide polymorphism frequencies and associations with recurrence‐free survival, cancer‐specific survival, and overall survival in intrahepatic cholangiocarcinoma.

SNP	*N* (%)	Recurrence‐free survival				Cancer‐specific survival				Overall survival			
		Median (95% CI)	*p* ^a^	HR (95% CI)	*p* ^b^	Median (95% CI)	*p* ^a^	HR (95% CI)	*p* ^b^	Median (95% CI)	*p* ^a^	HR (95% CI)	*p* ^b^
**Recessive model**													
*rs1047768*			0.468				0.711				0.386		
TT/TC	68 (66.6)	13 (8.5–17.5)		1		28 (14.7–41.3)		1		22 (12.0–32.0)		1	
CC	34 (33.4)	18 (5.1–30.9)		0.834 (0.506–1.376)	0.478	30 (17.1–42.9)		0.906 (0.537–1.531)	0.714	27 (14.1–39.9)		0.803 (0.486–1.326)	0.391
*rs1130409*			**0.022**				0.117				**0.046**		
TT/TG	90 (80.4)	15 (8.9–21.1)		1		29 (16.9–41.1)		1		27 (17.8–36.2)		1	
GG	22 (19.6)	7 (4.1–9.9)		1.929 (1.073–3.468)	0.028	25 (6.5–43.5)		1.571 (0.884–2.793)	0.124	16 (2.6–29.4)		1.694 (0.997–2.878)	0.051
*rs1805414*			0.740				0.118				**0.035**		
AA/AG	102 (91.1)	12 (7.1–16.9)		1		29 (19.3–38.7)		1		27 (18.3–35.7)		1	
GG	10 (8.9)	13 (7.1–18.9)		1.148 (0.496–2.657)	0.747	20 (6.7–33.3)		1.840 (0.839–4.036)	0.128	10 (5.4–14.6)		2.067 (1.028–4.157)	**0.042**
*rs2228001*			**0.044**				**0.034**				0.048		
GG/GT	71 (63.4)	9 (5.2–12.8)		1		24 (17.1–30.9)		1		20 (12.9–27.1)		1	
TT	41 (36.6)	20 (7.6–32.4)		0.619 (0.383–1.001)	0.051	50 (37.8–62.2)		0.580 (0.347–0.969)	**0.038**	46 (16.7–75.3)		0.626 (0.389–1.006)	0.053
*rs873601*			0.105				0.165				0.232		
GG/GA	60 (53.6)	13 (8.4–17.6)		1		29 (12.9–45.1)		1		24 (14.6–33.4)		1	
AA	51 (45.5)	10 (5.9–14.1)		1.436 (0.915–2.254)	0.115	27 (16.9–37.0)		1.389 (0.868–2.224)	0.171	25 (18.1–31.9)		1.302 (0.839–2.021)	0.239
**Codominant model**													
*rs1047768*			0.643				0.738				0.440		
TT	24 (23.5)	13 (4.1–21.9)		1		20 (0–55.7)				20 (8.5–31.5)			
TC	44 (43.1)	12 (8.5–15.5)		1.188 (0.654–2.158)	0.571	31 (14.7–47.3)		1.246 (0.653–2.376)	0.505	27 (12.0–41.9)		1.310 (0.727–2.361)	0.369
CC	34 (33.4)	18 (5.1–30.9)		0.928 (0.495–1.738)	0.815	30 (17.1–42.9)		1.044 (0.530–2.056)	0.902	27 (14.1–39.9)		0.955 (0.504–1.808)	0.887
*rs1130409*			0.067				0.256				0.121		
TT	31 (27.7)	19 (12.6–25.4)		1		28 (10.2–45.8)		1		27 (11.7–42.3)		1	
TG	59 (52.7)	13 (7.9–18.0)		1.110 (0.645–1.913)	0.706	30 (14.9–45.1)		0.872 (0.520–1.463)	0.605	29 (12.1–45.9)		0.860 (0.493–1.501)	0.596
GG	22 (19.6)	7 (4.1–9.9)		2.074 (1.030–4.179)	0.041	25 (6.5–43.5)		1.546 (0.824–2.899)	0.175	16 (2.6–29.4)		1.421 (0.719–2.805)	0.312
*rs1805414*			0.327				0.086				**0.026**		
AA	47 (42.0)	20 (12.8–27.2)		1		39 (26.2–51.8)		1		36 (23.1–48.9)		1	
AG	55 (49.1)	10 (7.3–12.7)		1.414 (0.878–2.278)	0.154	24 (14.6–33.4)		1.510 (0.943–2.416)	0.086	22 (12.4–31.6)		1.502 (0.906–2.491)	0.115
GG	10 (8.9)	13 (7.1–18.9)		1.383 (0.572–3.343)	0.472	20 (6.7–33.3)		2.606 (1.224–5.548)	0.013	10 (5.4–14.6)		2.311 (0.994–5.370)	0.052
*rs2228001*			**0.015**				**0.019**				0.066		
GG	19 (17.0)	17 (7.5–26.5)		1		42 (26.5–57.5)		1		38 (1.163–74.8)		1	
GT	52 (46.4)	7 (5.1–8.9)		1.903 (0.952–3.808)	0.069	20 (14.1–25.9)		1.447 (0.760–2.756)	0.261	20 (12.0–27.9)		1.838 (0.889–3.802)	0.101
TT	41 (36.6)	20 (7.6–32.4)		0.999 (0.479–2.081)	0.997	50 (37.8–62.2)		0.823 (0.413–1.640)	0.580	46 (16.7–75.3)		0.921 (0.421–2.012)	0.836
*rs873601*			0.208				0.332				0.279		
GG	11 (9.8)	15 (2.8–27.2)		1		28 (14.9–41.1)		1		18 (9.4–26.6)		1	
GA	49 (44.1)	13 (7.8–18.2)		1.437 (0.560–3.690)	0.451	36 (9.3–62.7)		0.655 (0.310–1.383)	0.267	30 (15.2–44.8)		0.775 (0.318–1.887)	0.574
AA	51 (45.9)	10 (5.9–14.1)		1.951 (0.767–4.962)	0.161	27 (16.9–37.0)		0.922 (0.443–1.917)	0.828	25 (18.1–31.9)		1.124 (0.474–2.663)	0.791
**Dominant model**													
*rs1047768*			0.835				0.645				0.631		
TT	24 (23.5)	13 (4.1–21.9)		1		20 (0–55.7)		1		20 (8.5–31.5)		1	
TC/CC	78 (76.5)	13 (7.4–18.6)		1.059 (0.613–1.829)	0.838	30 (20.5–39.5)		1.149 (0.633–2.087)	0.648	27 (19.7–34.3)		1.143 (0.660–1.979)	0.635
*rs1130409*			0.388				0.919				0.992		
TT	31 (27.7)	19 (12.6–25.4)		1		28 (10.2–45.8)		1		27 (11.7–42.3)		1	
GT/GG	81 (72.3)	11 (7.7–14.3)		1.252 (0.742–2.111)	0.400	28 (19.7–36.3)		1.003 (0.614–1.636)	0.992	25 (17.2–32.8)		0.973 (0.574–1.650)	0.919
*rs1805414*			0.135				0.060				**0.033**		
AA	47 (42.0)	20 (12.8–27.2)		1		39 (26.2–51.8)		1		36 (23.1–48.9)		1	
AG/GG	65 (58.0)	10 (6.9–13.1)		1.410 (0.887–2.241)	0.146	22 (13.4–30.6)		1.623 (1.030–2.557)	**0.037**	20 (12.9–27.1)		1.587 (0.972–2.592)	0.065
*rs2228001*			0.295				0.381				0.700		
GG	19 (17.0)	17 (7.5–26.5)		1		42 (26.5–57.5)		1		38 (1.2–74.8)		1	
GT/TT	93 (83.0)	10 (5.7–14.3)		1.414 (0.726–2.752)	0.308	27 (19.9–34.1)		1.127 (0.609–2.087)	0.703	25 (19.0–30.9)		1.363 (0.676–2.748)	0.387
*rs873601*			0.248				0.905				0.482		
GG	11 (9.8)	15 (2.8–27.2)		1		28 (14.9–41.1)		1		18 (9.4–26.6)		1	
GA/AA	100 (90.1)	11 (6.5–15.5)		1.676 (0.676–4.156)	0.265	28 (16.9–39.1)		0.781 (0.388–1.572)	0.488	27 (19.0–35.0)		0.951 (0.411–2.200)	0.906

*Note:* Bold values indicate statistical significane (*p* < 0.05).

^a^ Kaplan–Meier survival analysis.

^b^ Univariate Cox regression analyses.

### 3.3 Associations Between Patient Characteristics, SNPs and Outcomes

To meticulously examine the correlations between patient prognoses and SNPs, we employed both univariate and multivariate Cox regression analyses, incorporating clinical parameters alongside SNPs. Results of the univariate analysis are displayed in Table S3 while the multivariate analysis is summarised in Table 3. In our univariate analysis of RFS, we observed significant associations with neoadjuvant chemotherapy (*p* = 0.038), alkaline phosphatase (*p* = 0.026), haemoglobin (*p* = 0.048), C‐reactive protein (CRP, *p* = 0.019), intraoperative packed red blood cells (PRBCs, *p* = 0.022), intraoperative fresh frozen plasma (FFP, *p* = 0.034), microvascular invasion (MVI, *p* = 0.009), lymphovascular invasion (LVI, *p* < 0.001), tumour stage according to the Union for International Cancer Control (UICC, *p* = 0.003), nodal category (N category, *p* < 0.001), duration of hospitalisation (*p* = 0.006), and perioperative complications (*p* = 0.004) (Table S3). Additionally, certain genotypic distributions, such as *rs1130409* (GG vs. TT/GT, *p* = 0.028), and *rs1130409* (GG vs. TT, *p* = 0.041) exhibited significant correlations with RFS (Table S3). In the multivariable Cox regression model, variables with a *p*‐value < 0.1 were considered, excluding codominance and dominance models. Notably, LVI (hazard ratio [HR] = 2.601, *p* = 0.005), perioperative complications (HR = 1.920, *p* = 0.028), and *rs2228001* (TT vs. GG/GT, HR = 0.500, *p* = 0.027) emerged as independent prognostic factors for RFS (Table 3).

**TABLE 3:** Multivariable Cox regression analysis of recurrence‐free, cancer‐specific, and overall survival in intrahepatic cholangiocarcinoma.

Variables	Recurrence‐free survival		Cancer‐specific survival		Overall survival	
	HR (95% CI)	*p*	HR (95% CI)	*p*	HR (95% CI)	*p*
ASA (I/II =1)	—	—	—	—	2.409 (1.166–4.976)	0.017
Intraoperative PRBC (No = 1)	1.775 (1.088–2.896)	0.022	2.424 (1.225–4.796)	0.011	—	—
LVI (No = 1)	2.601 (1.331–5.081)	0.005	—	—	—	—
Tumour stage UICC (I/II = 1)	—	—	2.831 (1.654–4.843)	0.043	—	—
pT category (pT1‐2 = 1)	—	—	5.584 (1.650–18.898)	0.006	—	—
N category (pN0 = 1)	—	—	14.505 (2.798–75.201)	0.001	11.819 (2.172–64.296)	0.004
Perioperative complications (Clavien–Dindo) (0/I/II = 1)	1.920 (1.073–3.436)	0.028	—	—	—	—
rs1805414 (AA/AG =1)	—	—	—	—	4.307 (1.477–12.556)	0.007
rs2228001 (GG/GT = 1)	0.500 (0.271–0.923)	0.027	0.417 (0.202–0.861)	0.018	0.309 (0.142–0.672)	0.003

*Note:* Only significant parameters are shown. Abbreviations: ASA, American Society of Anesthesiology; HR, hazard ratio; LVI, lymph vascular invasion; MVI, microvascular invasion; PRBC, packed red blood cells.

In the context of CSS, pivotal clinical and pathological variables, such as albumin (*p* = 0.023), alkaline phosphatase (*p* = 0.023), gamma‐glutamyl transferase (GGT, *p* = 0.035), prothrombin time (*p* = 0.047), haemoglobin (*p* = 0.008), CRP (*p* = 0.001), intraoperative PRBCs (*p* = 0.009), intraoperative FFP (*p* = 0.005), MVI (*p* = 0.001), LVI (*p* = 0.001), UICC tumour stage (*p* < 0.001), pathological T category (*p* = 0.015), N category (*p* < 0.001), duration of hospitalisation (*p* = 0.023), and perioperative complications (*p* = 0.011) displayed significant associations in the univariate analysis (Table S3). Additionally, the presence of the SNP *rs2228001* (TT vs. GG/GT, *p* = 0.038) and *rs1805414* (GG vs. AA, *p* = 0.013) demonstrated a significant association (Table S3). All variables with a *p*‐value < 0.1 were included in a multivariable Cox regression model, once again excluding codominance and dominance models. Here, intraoperative PRBCs (HR = 2.424, *p* = 0.011), UICC tumour stage (HR = 2.831, *p* = 0.043), pT category (HR = 5.584, *p* = 0.006), N category (HR = 14.505, *p* = 0.001), and *rs2228001* (TT vs. GG/GT, HR = 0.417, *p* = 0.018) were identified as independent prognostic variables for CSS (Table 3).

Regarding OS, the ASA score (*p* = 0.018) and clinical markers such as albumin (*p* = 0.001), GGT (*p* = 0.021), alkaline phosphatase (*p* = 0.023), haemoglobin (*p* = 0.003), MVI (*p* = 0.005), LVI (*p* < 0.001), tumour grade (*p* = 0.012), IUCC tumour stage (*p* < 0.001), pT category (*p* = 0.014), N category (*p* < 0.001), hospitalisation (*p* = 0.018), perioperative complications (*p* = 0.012) and *rs1805414* (GG vs. AA/GA, *p* = 0.021) (Table S3) were significantly correlated with OS in univariate analysis. For the multivariable Cox regression model, variables with a *p*‐value < 0.1 were considered, excluding codominance and dominance models. Following multivariable analysis, variables such as ASA (HR = 2.409, *p* = 0.017), N category (HR = 11.819, *p* = 0.004), along with the genetic markers *rs1805414* (GG vs. AA/GA, HR = 4.307, *p* = 0.007), and *rs2228001* (TT vs. GG/GT, HR = 0.309, *p* = 0.003) were identified as independent prognostic variables (Table 3). Survival curves for *XPC rs2228001* are displayed in Figure 2A–C.

### 3.4 SNPs In Perihilar Cholangiocarcinoma

Genotype and allele frequencies for all five SNPs analysed in this study can be found in Table S2. All SNPs conform to the HWE. Among the SNPs examined, 11 patients with *rs1047768* (106/117) failed to be genotyped.

#### 3.4.1 Kaplan–Meier Analysis

After a median follow‐up period of 89 months, the study cohort exhibited median RFS, CCS and OS durations of 37, 49 and 33 months, respectively.

In the Kaplan–Meier survival analysis, the recessive models for *PARP1 rs1805414* exhibited statistical significance with RFS (*p* = 0.024). Specifically, the AA/AG genotypes of *PARP1 rs1805414* were associated with a median RFS of 42 months (95% CI = 21.6–62.4), in contrast to 21 months (95% CI = 3.0–39.3) for the GG genotype (*p* = 0.024).

Regarding CSS, the *XPC rs2228001* SNP demonstrated statistical significance in the recessive model (*p* = 0.021). The GT/GG genotypes exhibited a median CSS of 39 months (95% CI = 24.9–53.1), compared to a mean CSS of 88 months (95% CI = 68.8–108.6) for the TT genotype. No significant associations were observed between other SNPs and CSS. The results including univariate Cox regressions within all models are summarised in Table 4.

**TABLE 4:** Single nucleotide polymorphism frequencies and associations with recurrence‐free survival, cancer‐specific survival, and overall survival in perihilar cholangiocarcinoma.

SNP	*N* (%)	Recurrence‐free survival				Cancer‐specific survival				Overall survival			
		Median (95% CI)	*p* ^a^	HR (95% CI)	*p* ^b^	Median (95% CI)	*p* ^a^	HR (95% CI)	*p* ^b^	Median (95% CI)	*p* ^a^	HR (95% CI)	*p* ^b^
**Recessive model**													
*rs1047768*			0.755				0.495				0.386		
TT/TC	71 (67.0)	29 (11.2–46.8)		1		49 (19.3–78.7)		1		32 (21.0–43.0)		1	
CC	35 (33.0)	39 (0–86.0)		0.914 (0.519–1.611)	0.757	54 (11.9–96.1)		0.815 (0.452–1.471)	0.498	45 (17.6–72.4)		0.809 (0.499–1.313)	0.391
*rs1130409*			0.969				0.686				0.754		
TT/TG	94 (80.3)	35 (21.1–48.9)		1		49 (10.7–87.3)		1		31 (19.1–42.7)		1	
GG	23 (19.7)	45 (15.8–74.2)		0.988 (0.545–1.792)	0.969	50 (11.1–88.9)		1.127 (0.629–2.020)	0.688	45 (20.1–69.9)		0.921 (0.547–1.549)	0.756
*rs1805414*			**0.024**				0.121				0.056		
AA/AG	103 (88.0)	42 (21.6–62.4)		1		51 (31.5–70.5)		1		42 (32.9–60.0)		1	
GG	14 (12.0)	21 (3.0–39.0)		2.139 (1.079–4.242)	0.029	32 (27.4–36.6)		1.701 (0.858–3.374)	0.128	31 (26.1–35.9)		1.735 (0.973–3.093)	0.062
*rs2228001*			0.421				**0.021**				0.094		
GG/GT	82 (70.0)	29 (12.1–45.9)		1		39 (24.9–53.1)		1		30 (25.1–34.9)		1	
TT	35 (30.0)	52 (9.1–94.9)		0.801 (0.463–1.384)	0.426	89 (68.8–109)^c^		0.485 (0.258–0.913)	0.025	54 (46.3–61.7)		0.665 (0.410–1.080)	0.099
*rs873601*			0.110				0.818				0.566		
GG/GA	40 (34.2)	24 (0–52.8)		1		50 (5.4–94.6)		1		49 (25.0–72.9)		1	
AA	77 (65.8)	39 (24.7–53.3)		0.670 (0.406–1.104)	0.116	49 (27.1–70.8)		0.941 (0.560–1.580)	0.819	32 (20.8–43.2)		1.138 (0.728–1.779)	0.570
**Codominant model**													
*rs1047768*			0.916				0.222				0.438		
TT	26 (24.5)	36 (0–97.2)		1		25 (11.8–38.2)				19 (9.4–28.6)			
TC	45 (42.4)	29 (7.0–50.9)		0.910 (0.461–1.799)	0.787	65 (24.7–75.3)		0.599 (0.310–1.160)	0.129	38 (22.5–53.5)		0.775 (0.446–1.347)	0.366
CC	35 (33.0)	39 (0–86.0)		0.860 (0.421–1.757)	0.679	54 (11.9–96.1)		0.594 (0.296–1.192)	0.143	45 (17.6–72.4)		0.689 (0.383–1.239)	0.214
*rs1130409*			0.826				0.673				0.496		
TT	39 (33.3)	39 (0–81.1)		1		83 (22.2–143.8)		1		41 (16.1–65.9)		1	
TG	55 (47.0)	29 (15.3–42.7)		1.195 (0.674–2.121)	0.542	33 (6.5–59.5)		1.275 (0.702–2.316)	0.425	29 (22.0–35.9)		1.322 (0.811–2.156)	0.263
GG	23 (19.7)	45 (15.8–74.2)		1.093 (0.552–2.166)	0.798	50 (11.1–88.9)		1.296 (0.653–2.575)	0.459	45 (20.1–69.8)		1.082 (0.594–1.970)	0.798
*rs1805414*			0.073				0.271				0.119		
AA	59 (50.4)	42 (24.7–59.3)		1		51 (21.8–80.2)		1		41 (17.4–64.6)		1	
AG	44 (37.6)	35 (0–94.7)		1.126 (0.653–1.941)	0.669	54 (10.1–97.9)		1.143 (0.658–1.987)	0.634	27 (0.1–53.9)		1.207 (0.761–1.914)	0.425
GG	14 (12.0)	21 (3.0–39.0)		2.247 (1.090–4.631)	0.028	32 (27.4–36.6)		1.801 (0.871–3.727)	0.113	31 (26.1–35.9)		1.884 (1.017–4.387)	0.044
*rs2228001*			0.627				0.070				0.117		
GG	21 (17.9)	42 (20.5–63.5)		1		41 (0–95.4)		1		41 (0.5–81.5)		1	
GT	61 (52.1)	28 (13.6–42.4)		1.195 (0.604–2.364)	0.608	33 (15.2–50.8)		1.036 (0.548–1.958)	0.914	29 (24.2–33.8)		1.410 (0.778–2.554)	0.258
TT	35 (29.9)	52 (9.1–94.9)		0.913 (0.431–1.935)	0.812	49 (23.7–74.3)		0.498 (0.227–1.092)	0.082	54 (46.3–61.7)		0.861 (0.440–1.685)	0.662
*rs873601*			0.256				0.972				0.738		
GG	3 (2.6)	55 (50.2–59.8)		1		84 (50.4–117.6)		1		84 (50.4–117.6)		1	
GA	37 (31.6)	24 (11.7–36.3)		1.439 (0.561–3.694)	0.449	49 (13.2–84.8)		1.033 (0.306–3.498)	0.959	38 (8.5–67.5)		1.394 (0.420–4.628)	0.588
AA	77 (65.8)	39 (24.7–53.3)		1.954 (0.768–4.970)	0.159	49 (27.2–70.8)		0.968 (0.297–3.155)	0.957	32 (20.8–41.2)		1.525 (0.475–4.893)	0.478
**Dominant model**													
*rs1047768*			0.705				0.083				0.225		
TT	26 (24.5)	36 (0–97.2)		1		25 (11.8–38.2)		1		19 (9.4–28.6)		1	
TC/CC	80 (75.4)	39 (20.9–57.1)		0.888 (0.475–1.657)	0.708	54 (25.7–82.3)		0.597 (0.330–1.079)	0.088	45 (29.3–40.7)		0.735 (0.445–1.217)	0.232
*rs1130409*			0.583				0.374				0.356		
TT	39 (33.3)	39 (0–81.1)		1		83 (22.2–143.8)		1		41 (16.1–65.9)		1	
GT/GG	78 (66.7)	35 (15.5–54.5)		1.160 (0.681–1.976)	0.586	49 (27.2–70.8)		1.283 (0.737–2.231)	0.378	32 (21.7–42.3)		1.239 (0.783–1.960)	0.361
*rs1805414*			0.258				0.324				0.162		
AA	59 (50.4)	42 (24.7–59.3)		1		51.0 (21.8–80.2)		1		41 (17.4–64.6)		1	
AG/GG	58 (49.6)	24 (4.2–43.8)		1.326 (0.808–2.176)	0.264	45 (21.7–68.3)		1.286 (0.777–2.129)	0.328	31 (21.0–40.9)		1.348 (0.882–2.059)	0.167
*rs2228001*			0.818				0.510				0.568		
GG	21 (17.9)	42 (20.5–63.5)		1		41 (0–95.4)		1		41 (0.5–81.5)		1	
GT/TT	96 (82.1)	35 (15.6–54.4)		1.079 (0.562–2.071)	0.820	50 (26.9–73.1)		0.814 (0.441–1.505)	0.513	33 (21.0–44.9)		1.180 (0.665–2.094)	0.572
*rs873601*			0.928				0.985				0.499		
GG	3 (2.6)	55 (50.2–59.8)		1		84 (50.4–117.6)		1		84 (50.4–117.6)		1	
GA/AA	114 (97.4)	36 (21.8–50.2)		0.948 (0.296–3.036)	0.929	49 (31.6–66.4)		0.989 (0.308–3.178)	0.985	32 (21.6–42.4)		1.481 (0.466–4.711)	0.506

*Note:* Bold values indicate statistical significane (*p* < 0.05).

^a^ Kaplan–Meier survival analysis.

^b^ Univariate Cox regression analyses.

^c^ Mean.

### 3.5 Associations Between Patient Characteristics, SNPs and Outcomes

To elucidate the associations between patient outcomes and potential prognostic factors, we conducted both univariate and multivariate Cox regression analyses, incorporating clinical parameters and SNPs. In the univariate analysis for RFS, significant associations were observed with haemoglobin levels (*p* < 0.001), operative time (*p* = 0.018), intraoperative PRBC transfusion (*p* = 0.017), intraoperative FFP transfusion (*p* = 0.007), MVI (*p* = 0.042), LVI (*p* = 0.017), tumour grade (*p* < 0.001), UICC tumour stage (*p* = 0.021), pT category (*p* = 0.012), N category (*p* = 0.002), adjuvant therapy (*p* = 0.044) (Table S4), and SNP *rs1805414* in both comparison models (GG vs. AA/GA, *p* = 0.029; GG vs. AA, *p* = 0.028) (Table S4). For the multivariate Cox regression model, all variables demonstrating a *p*‐value < 0.1 were included, with the exclusion of codominant and dominant genetic models. Here, haemoglobin (HR = 0.314, *p* < 0.001), intraoperative FFP (HR = 2.796, *p* = 0.002), MVI (HR = 66.350, *p* < 0.001), tumour grade (HR = 3.301, *p* = 0.003), N category (HR = 2.829, *p* = 0.001), and SNP *rs1805414* (GG vs. AA/GA, HR = 3.909, *p* = 0.005) were identified as independent prognostic variables for RFS (Table 5).

**TABLE 5:** Multivariable Cox regression analysis of recurrence‐free, cancer‐specific, and overall survival in perihilar cholangiocarcinoma.

Variables	Recurrence‐free survival		Cancer‐specific survival		Overall survival	
	HR (95% CI)	*p*	HR (95% CI)	*p*	HR (95% CI)	*p*
Haemoglobin, g/L (≤ 12 = 1)	0.314 (0.166–0.592)	< 0.001	0.334 (0.175–0.638)	< 0.001	0.385 (0.226–0.654)	< 0.001
Intraoperative FFP (No =1)	2.796 (1.453–5.381)	0.002	4.262 (2.045–8.881)	< 0.001	3.072 (1.762–5.354)	< 0.001
MVI (No = 1)	66.350 (11.621–378.813)	< 0.001	40.523 (6.944–236.477)	< 0.001	19.083 (3.880–93.844)	< 0.001
Tumour grading (G1/G2 = 1)	3.301 (1.482–7.352)	0.003	5.420 (2.406–12.213)	< 0.001	3.699 (1.879–7.284)	< 0.001
pT category (pT1‐2 = 1)	—	—	2.291 (1.192–4.405)	0.013	2.227 (1.309–3.789)	0.003
N category (pN0 = 1)	2.829 (1.511–5.296)	0.001	2.638 (1.408–4.941)	0.002	1.779 (1.055–2.998)	0.031
Perioperative complications (Clavien–Dindo) (0/I/II =1)	—	—	1.911 (1.030–3.546)	0.040	2.115 (1.279–3.497)	0.004
rs1805414 (AA/AG =1)	3.909 (1.517–10.076)	0.005	—	—	—	—
rs2228001 (GG/GT = 1)	—	—	0.475 (0.233–0.971)	0.041	—	—

*Note:* Only significant parameters are shown. Abbreviations: BMI, Body mass index; CSS, cancer‐specific survival; FFP, fresh frozen plasma; MVI, microvascular invasion; OS, Overall Survival; PRBC, packed red blood cells.

In the univariate analysis for CSS, associations were observed with international normalised ratio (*p* = 0.023), haemoglobin levels (*p* = 0.012), intraoperative PRBC transfusion (*p* = 0.003), intraoperative FFP transfusion (*p* < 0.001), MVI (*p* = 0.013), LVI (*p* = 0.043), tumour grade (*p* < 0.001), UICC tumour stage (*p* = 0.008), pT category (*p* = 0.002), N category (*p* < 0.001), perioperative complications (*p* = 0.033) and *SNP rs2228001* (TT vs. GG/GT, *p* = 0.011) (Table S4). In the multivariate Cox regression model, variables with *p*‐values < 0.1 were included, again excluding codominance and dominance genetic models. Independent prognostic factors for CSS included haemoglobin (HR = 0.334, *p* < 0.001), intraoperative FFP (HR = 4.262, *p* < 0.001), pT category (HR = 2.291, *p* = 0.013), N category (HR = 2.638, *p* = 0.002), perioperative complications (HR = 1.911, *p* = 0.040), and *SNP rs2228001* (TT vs. GG/GT, HR = 0.475, *p* = 0.041) (Table 5).

For OS, univariate analysis revealed significant associations with haemoglobin (*p* < 0.001), intraoperative PRBC transfusion (*p* < 0.001), intraoperative FFP (*p* < 0.001), MVI (*p* = 0.046), LVI (*p* = 0.003), tumour grade (*p* < 0.001), UICC tumour stage (*p* = 0.040), pT category (*p* = 0.002), N category (*p* = 0.007), duration of ICU stay (*p* = 0.021), and perioperative complications (*p* = 0.004) (Table S4). For the multivariate Cox regression model, variables with *p*‐values < 0.1 were included, with the exclusion of codominance and dominance genetic models. In the multivariate model, haemoglobin (HR = 0.385, *p* < 0.001), intraoperative FFP (HR = 3.072, *p* < 0.001), MVI (HR = 19.083, *p* < 0.001), tumour grade (HR = 3.699, *p* < 0.001), pT category (HR = 2.227, *p* = 0.003), N category (HR = 1.779, *p* = 0.031), and perioperative complications (HR = 2.115, *p* = 0.004) emerged as independent prognostic factors for OS (Table 5). Survival curves for *XPC rs2228001* are displayed in Figure 2D–F.

### 3.6 Stratified Survival Analysis According to Adjuvant Chemotherapy Application

To evaluate whether the prognostic impact of SNPs was influenced by adjuvant chemotherapy, we performed subgroup analyses stratified by adjuvant treatment status in iCCA and pCCA (Tables S5–, S8). In iCCA patients who did not receive adjuvant therapy, the TT genotype of XPC rs2228001 showed a trend towards improved RFS, CSS and OS compared to GG/GT genotypes, although statistical significance was not reached (RFS: HR = 0.676, *p* = 0.219; CSS: HR = 0.730, *p* = 0.328; OS: HR = 0.756, *p* = 0.332). In contrast, in iCCA patients who received adjuvant chemotherapy, the TT genotype of rs2228001 was associated with better outcomes, particularly for CSS (HR = 0.398, *p* = 0.066) and OS (HR = 0.398, *p* = 0.066).

In pCCA patients without adjuvant chemotherapy, the TT genotype also indicated a favourable trend for CSS and OS, but did not reach statistical significance (CSS: HR = 0.517, *p* = 0.092; OS: HR = 0.695, *p* = 0.218). Interestingly, in pCCA patients who received adjuvant therapy, the TT genotype of XPC rs2228001 was significantly associated with longer CSS (HR = 0.292, *p* = 0.037) and OS (HR = 0.338, *p* = 0.028) when compared to the GG/GT genotypes.

### 3.7 Combined Single Nucleotide Burden and Survival Outcomes

To evaluate whether the cumulative burden of DNA repair SNPs impacts clinical outcomes, patients were stratified based on the number of risk‐associated genotypes (≤ 3 vs. > 3). In iCCA (Figure S1A–C), no statistically significant differences were observed in RFS (*p* = 0.940), CSS (*p* = 0.985), or OS (*p* = 0.971) between the two groups. Similarly, in pCCA (Figure S1D–F), patients with > 3 risk SNPs did not show significantly different RFS (*p* = 0.107), CSS (*p* = 0.341) or OS (*p* = 0.948) compared to those with ≤ 3 variants. These findings suggest that the prognostic impact of individual SNPs may not be additive when considered in combination.

## 4 Discussion

CCA is a highly lethal malignancy, which is often diagnosed at advanced stages due to its unspecific onset [19]. The insidious nature of CCA often limits treatment to palliative care, which provides minimal clinical benefit. Although SNPs have been extensively studied in various malignancies, their association with CCA has remained elusive and warrants further investigation. In this study, we comprehensively analysed 5 SNPs located in gene loci associated with DNA repair. Here, SNPs (*rs2228001*) in the DNA repair gene *XPC* exhibited a strong association with RFS, CSS and OS in iCCA and CSS in pCCA. Moreover, *PARP1 SNP rs1805414* demonstrated prognostic value correlating with OS in iCCA and RFS in pCCA.

Variability in DNA damage repair (DDR) capacity plays a pivotal role in cancer progression and has emerged as a novel therapeutic target in oncology [20]. In every cell, DNA repair pathways exist to maintain genomic integrity [21]. Furthermore, polymorphisms within DNA repair genes are recognised as factors that influence individual susceptibility to cancer [22, 23, 24]. The European Network for the Study of CCA suggests that DDR pathways are significantly associated with CCA development and might also contribute to chemotherapy resistance in CCA [24].

The XPC gene, pivotal in Nucleotide Excision Repair (NER) plays a vital role in mitigating DNA damage [25]. Decreased or absent XPC expression leads to elevated levels of intracellular reactive oxygen species, increasing oxidative stress and subsequent oxidative DNA damage [26]. Mutations in the *XPC* gene can compromise its NER capabilities, potentially initiating carcinogenesis [27]. The XPC‐HR23B complex has been demonstrated to recognise DNA lesions, stimulate the activity of 8‐Oxoguanine glycosylase (OGG1) and constrain the efficacy of NER [28]. Numerous studies have associated SNPs in the *XPC* gene with various cancer types, including breast and colorectal cancers [29]. Located on chromosome 3p25.1, the *XPC* gene is composed of 15 introns and 16 exons. The SNP *rs2228001* is characterised by a C‐to‐A substitution at cDNA position 2815 and is the most extensively investigated SNP in this gene locus [30]. Recent findings have revealed a significant correlation between the substitution at *XPC* 939 and a higher incidence of p53 mutations in aflatoxin B1‐induced HCC (AFB1), illustrating *XPC's* significant role in mitigating AFB1‐induced genotoxic effects [31, 32]. This specific polymorphism, *rs2228001*, has been associated with an increased risk of cervical cancer and greater tumour aggressiveness in a Bangladeshi population [33]. *Rs2228001* has equally been suggested to possibly influence *XPC*'s functionality and modulate the risk of various other cancers [34, 35]. In our analysis, the TT genotype of *XPC rs2228001* was associated with a significant prolongation of RFS, CSS and OS in iCCA, compared to the GG/GT genotype. Furthermore, multivariable Cox regression, including a large set of clinical and analysis, identified the TT genotype of *XPC rs2228001* as an independent prognostic indicator for RFS, CSS and OS in iCCA and CSS in pCCA.

This notable clinical association underlines the critical role of DNA repair pathways in the maintenance of genomic stability, genomic instability, and implications in tumour progression. One plausible explanation for this observation might be a role of the XPC protein in initiating NER. In individuals with impaired XPC function due to genetic variations, the accumulation of DNA damage may be accelerated, leading to increased genomic instability, a well‐known driver of cancer recurrence and progression after surgical intervention [36]. Moreover, residual tumour cells left after surgery are often exposed to post‐operative stressors, such as hypoxia, which can further exacerbate DNA damage. In patients with compromised DNA damage repair due to the *rs2228001* polymorphism, this could lead to a higher likelihood of tumour recurrence.

Another relevant SNP identified in our analysis was *rs1805414* within the Poly (ADP‐Ribose) Polymerase 1(PARP‐1) Protein. PARP‐1 is a nuclear protein which is highly expressed within cells and constitutes a crucial component of the DNA Base Excision Repair (BER) system, as well as an essential part of NER and the classical Non‐Homologous End Joining (NHEJ) pathway [37, 38, 39]. PARP‐1 acts as an enzyme playing a notable role in DNA damage repair, differentiation and proliferation [40, 41]. Upon DNA damage, PARP‐1 is activated and recruited to the sites of DNA injury, catalysing the cleavage of Nicotinamide adenine dinucleotide (NAD+) into nicotinamide and ADP‐ribose [42, 43]. This reaction results in the formation of elongated branches of ADP‐ribose polymers capable of modifying target proteins. However, excessive activation of PARP can deplete NAD+ and ATP, thereby compromising cellular energy supply and potentially inducing necrotic cell death [44]. The SNP *rs1805414* is characterised by a C‐to‐A substitution at cDNA position 852 within exon 7 of *PARP1* and has been associated with an increased risk of breast cancer but a decreased risk of colorectal cancer [45, 46, 47]. Our investigation revealed that the *PARP1 rs1805414* GG genotype is associated with a reduction in OS in iCCA and decreased RFS in pCCA. The *rs1805414*‐related region is associated with the promoter of *PARP‐1* [48]. Given that the regulation of the *PARP1* gene hinges on its promoter, alterations in the transcription binding sites within this region may negatively affect its expression, thereby reducing protein abundance and contributing to the progression of cancer [48]. In addition, the *PARP1 rs1805414* polymorphism, though less studied, is known to influence the efficacy of single‐strand break repair (SSBR) through the PARP1 enzyme, which is essential for repairing single‐strand DNA breaks. Deficient SSBR can lead to an accumulation of single‐strand breaks, which, if not repaired, may convert to double‐strand breaks, further compromising genomic stability allowing for the accumulation of mutations to promote cancer recurrence and progression [49, 50].

Recently, PARP‐1 inhibitors have been introduced to the armamentarium of anti‐cancer drugs with the concept of rendering tumours more susceptible to single‐strand DNA breaks caused by chemotherapy and radiotherapy [51, 52]. While PARP‐1 inhibitors have currently no role in the treatment of CCA, the potential influence of SNPs on PARP‐1 function does implicate further investigations in the interaction of SNPs, PARP activity and PARP‐1 inhibitors.

While not as pronounced as the previously mentioned SNPs, which have shown significance in a multivariable Cox regression model, SNPs on the *APEX1* gene were associated with RFS and OS in iCCA within the univariate Kaplan–Meier analysis. *APEX1*, located on chromosome 14q11.2 and comprising five exons spanning approximately 2.5–3 kb of DNA, not only plays a significant role in DNA damage repair through the BER pathway but also functions as a transcriptional co‐activator for multiple transcription factors, such as p53 and NF‐κB, which are integral in oncogenesis and cancer progression [53, 54]. *APEX1* also presents a potential target for drug therapy in certain cancer types [55]. Additionally, serving as a multifunctional protein, APEX1 participates in essential cellular processes, including oxidative stress response, regulation of transcription factors, cell cycle control, and apoptosis. Thus, aberrant expression of the *APEX1* gene may compromise the proper functioning of vital cellular processes, potentially elevating an individual's susceptibility to cancer [56, 57, 58]. *APEX1* is characterised by a high degree of polymorphism, with 18 reported polymorphisms in the literature [59]. Among these, *rs1130409* stands out as a prevalent polymorphism potentially influencing its endonuclease and DNA binding activities [60]. This polymorphism could alter the structural configuration of the APEX1 protein, reducing its repair efficiency, and has been associated with various cancer types [59, 61]. The T‐to‐G codon polymorphism in *APEX1* is correlated with delayed mitosis in lymphocytes of healthy participants, suggesting increased sensitivity to ionising radiation, with the G allele predisposing to more significant genetic damage [62].

In the context of CCA, where high post‐surgical recurrence rates are common, the ability of cancer cells to repair DNA damage is crucial for preventing further mutations and recurrence. SNPs that impair DNA repair capacity, therefore, may exacerbate the genetic instability of residual tumour cells, increasing the chances of recurrence and leading to poorer survival outcomes. Our findings provide further support for the notion that SNPs in DNA repair genes, particularly in the *XPC* and *PARP1* genes, may serve as prognostic biomarkers for patients with CCA, and highlight the importance of the DNA repair pathways in modulating tumour biology after surgical resection.

Based on our findings, a potential clinical pathway for the application of XPC rs2228001 as a prognostic biomarker could involve pre‐operative genotyping to stratify patients according to genetic risk. Specifically, patients carrying the TT genotype, which was associated with favourable recurrence‐free and overall survival, may be managed with standard post‐operative surveillance protocols. In contrast, patients harbouring the GG or GT genotypes, linked to poorer outcomes, could benefit from an intensified post‐operative monitoring strategy. This may include more frequent imaging assessments (CT or MRI), shorter follow‐up intervals, and a more aggressive evaluation for adjuvant therapies or inclusion in clinical trials. Furthermore, genotyping could be incorporated into multidisciplinary tumour board discussions to inform personalised therapeutic decisions, including the consideration of neoadjuvant or novel targeted therapies in high‐risk individuals. Prospective validation of such genotype‐based stratification pathways is warranted to ensure clinical utility and integration into cholangiocarcinoma management guidelines.

Consistent with observations across multiple malignancies, SNPs within DNA repair pathways have emerged as critical determinants of oncological outcomes. In colorectal cancer, for example, polymorphisms in genes such as *XRCC1* and *ERCC1* have been linked to variations in recurrence‐free and overall survival, underscoring their prognostic relevance [63]. Similarly, extensive investigations in breast cancer have demonstrated that alterations within *BRCA1* and *BRCA2* not only modulate cancer susceptibility but also significantly influence survival trajectories [64]. In lung cancer, SNPs in genes such as *XPD* and *XRCC3* have been consistently implicated as key predictors of therapeutic response and prognosis [65]. In this context, our study extends these insights by demonstrating that the XPC rs2228001 variant confers significant prognostic implications in cholangiocarcinoma, highlighting the pivotal role of DNA repair capacity in shaping tumour progression and patient survival across diverse cancer types.

We conducted stratified analyses to determine whether adjuvant chemotherapy modified the prognostic relevance of DNA repair gene SNPs. While the overall trends in both treated and untreated subgroups mirrored those of the full cohort, the prognostic strength of the TT genotype of XPC rs2228001 appeared particularly pronounced among patients who received adjuvant chemotherapy. This was especially evident in pCCA, where TT carriers showed significantly improved cancer‐specific and overall survival under adjuvant treatment. These findings support the hypothesis that DNA repair capacity, modulated by germline variation, may alter sensitivity to DNA‐damaging agents such as cisplatin and gemcitabine. The enhanced outcomes in TT genotype carriers might reflect improved response to chemotherapy or better inherent repair efficiency. Although these observations require validation in larger cohorts, they further highlight the potential of incorporating SNP genotyping into pre‐treatment stratification and tailoring adjuvant regimens based on patient genetic profiles.

We further explored whether the cumulative number of unfavourable SNPs could influence survival outcomes by comparing patients with ≤ 3 versus > 3 risk genotypes. However, no statistically significant differences in RFS, CSS, or OS were observed in either iCCA or pCCA subgroups. This finding indicates that, at least within our cohort, the prognostic impact of individual SNPs such as XPC rs2228001 and PARP1 rs1805414 appears to act independently rather than synergistically. The lack of additive effect may reflect the complexity of DNA repair networks, wherein compensatory mechanisms or pathway redundancies may mitigate the influence of multiple polymorphisms. Alternatively, insufficient statistical power may have limited the detection of subtle combined effects. Future studies employing multi‐locus interaction models in larger, multi‐institutional datasets are warranted to clarify whether polygenic risk scores or SNP combinations could enhance prognostic stratification in CCA.

Although the present study focused primarily on the prognostic implications of SNPs in cholangiocarcinoma, emerging evidence suggests that genetic polymorphisms may also influence the risk of CCA development. For example, a case–control study by Sun et al. reported that polymorphisms in ERCC1 and XPF genes were associated with an increased risk of extrahepatic cholangiocarcinoma in a Chinese population [23]. Similarly, research investigating DNA repair genes such as XRCC1 and OGG1 has suggested potential associations with CCA susceptibility, although findings remain inconsistent across studies and populations. These observations align with the established role of impaired DNA repair mechanisms in carcinogenesis. However, current literature on the direct association between the specific SNPs investigated in this study and cholangiocarcinoma risk remains scarce. Further large‐scale, population‐based studies are needed to elucidate whether these variants contribute to CCA development in addition to modulating disease progression and prognosis.

The clinical implications of our findings are noteworthy. SNPs in DNA repair genes, such as *XPC rs2228001* and *PARP1 rs1805414*, can serve as valuable biomarkers for guiding pre‐operative risk assessment and post‐operative surveillance in CCA patients. Given that these genetic variations are associated with poorer survival outcomes, identifying patients with unfavourable SNP profiles pre‐operatively may enable clinicians to stratify patients according to risk, allowing for more tailored surgical and adjuvant treatment plans. Furthermore, patients with high‐risk SNP profiles may benefit from closer surveillance during follow‐up, as they are more likely to experience tumour recurrence due to impaired DNA repair mechanisms. Early detection of recurrence through intensified monitoring could potentially improve overall survival by allowing for timely interventions. One of the key advantages of SNP analysis is its technical simplicity and accessibility. Since SNPs are germline variants, genotyping can be performed on genomic DNA extracted from peripheral blood mononuclear cells (PBMCs), requiring only a minimally invasive blood sample. This straightforward approach enables cost‐effective and reliable testing without the need for tumour tissue. The ease of integration into routine clinical workflows makes SNP testing a particularly attractive tool for personalised medicine. In the future, SNP screening could be incorporated into standard pre‐operative risk stratification protocols for patients with CCA, supporting individualised treatment strategies based on host genetic profiles.

Considering these findings, future clinical trials should focus on validating the utility of SNP genotyping for risk stratification and follow‐up management in larger cohorts. Additionally, exploring potential therapeutic interventions that target DNA repair pathways may offer new opportunities to improve outcomes in patients with unfavourable SNP profiles, particularly those identified at higher risk for recurrence.

The study exhibits several limitations worth noting. It is retrospective in nature and conducted at a single hepatobiliary centre, potentially limiting the generalisability of the results. While the sample size is considerable, validating the findings across multiple centres or countries would bolster their applicability. Although the array of examined SNPs is extensive, it is not exhaustive in capturing all DNA repair SNPs potentially associated with CCA. Furthermore, the observed associations do not establish a definitive causal link between specific SNPs and CCA. This limitation requires future in vitro analysis to investigate the biological mechanisms behind our findings. This also includes the functional consequences of the investigated SNPs in CCA tumour tissue, such as gene or protein expression levels or repair activity. This limitation precludes definitive conclusions about whether these variants represent loss‐ or gain‐of‐function mutations. Future studies combining SNP analysis with transcriptomic and proteomic profiling are needed to elucidate the phenotypic impact of these polymorphisms. Also, assessments between mutational status and SNP characteristics would be worth pursuing; however, this data is not available within our data set. Moreover, the scope of our research was limited to patients who underwent surgical resection for iCCA and pCCA. As such, the conclusions derived from our data are not applicable to broader populations of patients with iCCA or pCCA who are ineligible for surgery, particularly those with locally advanced or metastatic disease. Further, dCCA was not included in this analysis, as suitable tissue for genomic evaluation was not available in sufficient quality and quantity for this subgroup. Therefore, the present findings are limited to iCCA and pCCA, and caution is advised when generalising results to dCCA.

## Author Contributions

Conceptualization, G.W., A.M. and J.B.; methodology, G.W., S.S.L., J.S., L.R.H. and J.B.; software, G.W., A.M. and J.B.; validation, D.L. and T.A.M.; formal analysis, G.W., A.M., D.L., T.A.M., U.P.N., L.R.H., J.B.; investigation, G.W., A.M., D.L., T.A.M., S.S.L., J.S., T.L., T.F.U., I.A., F.W.R.V., G.L., U.P.N., L.R.H. and J.B.; resources, U.P.N. and J.B.; data curation, D.L. and T.A.M.; writing – original draft preparation, G.W., A.M. and J.B.; writing – review and editing, D.L., T.A.M., S.S.L., J.S., T.L., T.F.U., I.A., F.W.R.V., G.L., U.P.N. and L.R.H.; visualisation, G.W. and A.M.; supervision, J.B. and U.P.N.; project administration, L.R.H. and J.B.; funding acquisition, G.W., D.L. and J.B. All authors have read the manuscript and are in agreement with the content.

## Ethics Statement

This study was conducted in accordance with the current version of the Declaration of Helsinki and good clinical practice guidelines (International Conference on Harmonisation, Good Clinical Practice). Informed consent was obtained from the included patients. Approval was granted by the institutional review board (Independent Ethics Committee, RWTH University Aachen, Germany, EK 252/15, EK 206/09).

## Conflicts of Interest

The authors declare no conflicts of interest.

## Supporting information


**Figure S1:** Oncological outcomes in relation to cumulative occurrence single nucleotide polymorphisms.Patients were stratified based on the number of non‐wild type genotypes (≤ 3 vs. > 3). A–C: RFS (A), CCS (B) and OS (C) in iCCA. D‐F: RFS (D), CCS (E) and OS (F) in pCCA. CCS, cancer‐specific survival; iCCA, intrahepatic cholangiocarcinoma; OS, overall survival; pCCA, perihilar cholangiocarcinoma; RFS, recurrence‐free survival.

**Table S1:** Genes and their single nucleotide polymorphism sequences.

**Table S2:** Polymorphisms, genotypes, allele frequencies and Hardy–Weinberg Equilibrium.

**Table S3:** Patient characteristics in association with RFS, CSS and OS in iCCA.

**Table S4:** Patient characteristics in association with RFS, CSS and OS in pCCA.

**Table S5:** Single nucleotide polymorphism frequencies and associations with recurrence‐free survival, cancer‐specific survival and overall survival in intrahepatic cholangiocarcinoma without adjuvant therapy.

**Table S6:** Single nucleotide polymorphism frequencies and associations with recurrence‐free survival, cancer‐specific survival and overall survival in intrahepatic cholangiocarcinoma with adjuvant therapy.

**Table S7:** Single nucleotide polymorphism frequencies and associations with recurrence‐free survival, cancer‐specific survival and overall survival in perihilar cholangiocarcinoma without adjuvant therapy.

**Table S8:** Single nucleotide polymorphism frequencies and associations with recurrence‐free survival, cancer‐specific survival and overall survival in perihilar cholangiocarcinoma with adjuvant therapy.

## Data Availability

The data that support the findings of this study are available from the corresponding author upon reasonable request.
