# Supplementary figures and images for: The Prognostic Value of the XPC rs2228001 Single Nucleotide Polymorphism in Cholangiocarcinoma
Source: Liver Int. 2025 Aug 20;45(9):e70292. doi: 10.1111/liv.70292 (PMC12366541; doi:10.1111/liv.70292)

**A**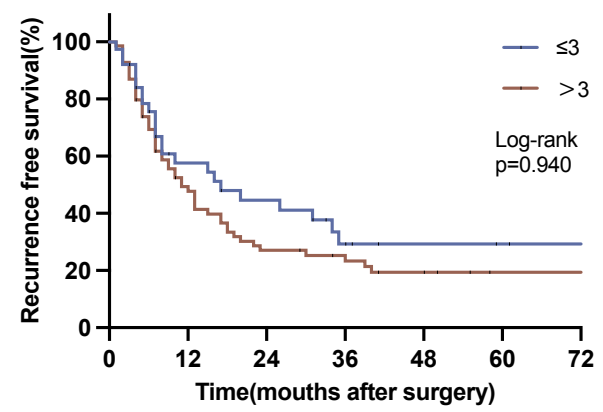**D**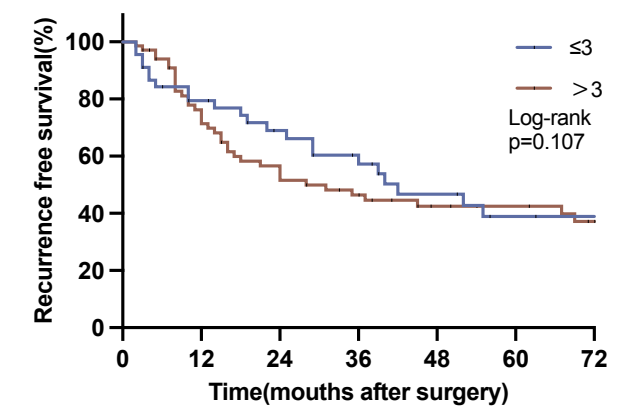**B**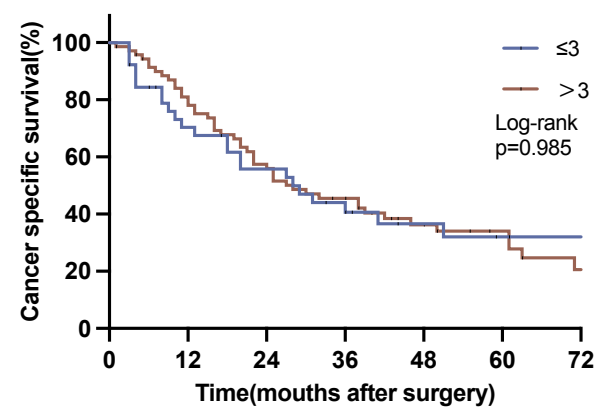**E**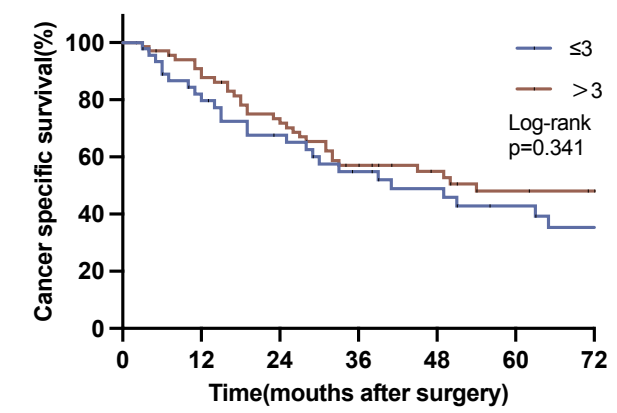**C**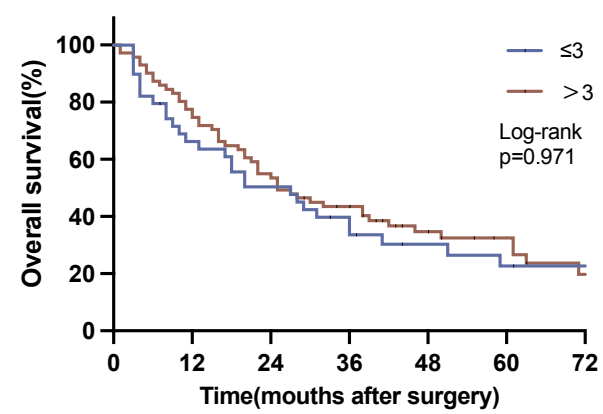**F**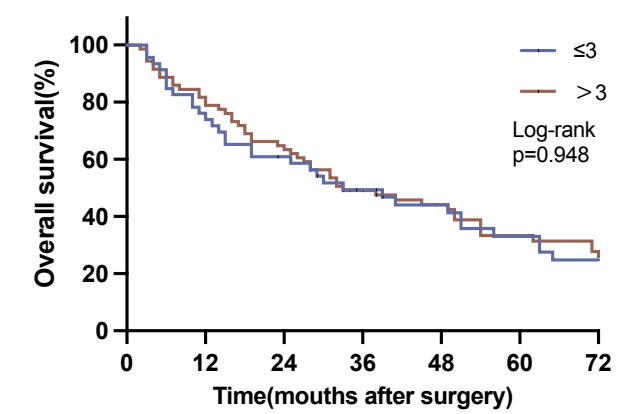

Supplement: Supplementary file 1 — Figure S1: Oncological outcomes in relation to cumulative occurrence single nucleotide polymorphisms.Patients were stratified based on the number of non‐wild type genotypes (≤ 3 vs. > 3). A–C: RFS (A), CCS (B) and OS (C) in iCCA. D‐F: RFS (D), CCS (E) and OS (F) in pCCA. CCS, cancer‐specific survival; iCCA, intrahepatic cholangiocarcinoma; OS, overall survival; pCCA, perihilar cholangiocarcinoma; RFS, recurrence‐free survival. [file LIV-45-0-s004.pdf]
