# Supplementary material for: The Prognostic Value of the XPC rs2228001 Single Nucleotide Polymorphism in Cholangiocarcinoma
Source: Liver Int. 2025 Aug 20;45(9):e70292. doi: 10.1111/liv.70292 (PMC12366541; doi:10.1111/liv.70292)
Supplement: Supplementary file 2 — Table S1: Genes and their single nucleotide polymorphism sequences. [file LIV-45-0-s008.docx]

**Supplementary table S1. Genes and their single nucleotide polymorphism sequences**

| **SNP** | **Gene** | **Location** | **Context Sequence[VIC/FAM]** |
| --- | --- | --- | --- |
| *rs1047768* | *ERCC5* | Chr.13: 102852167 | CACTTAAAGGAGTCCGGGATCGCCA [C/T] GGGAACTCAATAGAAAATCCTCATC |
| rs1130409 | *APEX1* | Chr.14: 20456995 | AATTCTGTTTCATTTCTATAGGCGA[G/T]GAGGAGCATGATCAGGAAGGCCGGG |
| rs1805414 | *PARP1* | Chr.1: 226385663 | GGAGGGCACCGAACACCATGCCATC[G/A]GCTACTCGGTCCAAGATCTGCAGCC |
| rs2228001 | *XPC* | Chr.3: 14145949 | TCTAGTGGGCGCTCAGCTCACAGCT[G/T]CTCAAATGGGAACAGGTGGGAAGCT |
| rs873601 | *ERCC5* | Chr.13: 102875987 | CAAAGACGTAATAAAATTAACTGGT[G/A]GCACGGTCTTTGTATTTAGTGTGTG |

APEX1, apurinic/apyrimidinic endodeoxyribonuclease 1; ERCC5, ERCC excision repair 5; PARP1, poly(ADP-ribose) polymerase 1; XPC, Xeroderma pigmentosum complementation group C.
