# Supplementary material for: The Prognostic Value of the XPC rs2228001 Single Nucleotide Polymorphism in Cholangiocarcinoma
Source: Liver Int. 2025 Aug 20;45(9):e70292. doi: 10.1111/liv.70292 (PMC12366541; doi:10.1111/liv.70292)
Supplement: Supplementary file 3 — Table S2: Polymorphisms, genotypes, allele frequencies and Hardy–Weinberg Equilibrium. [file LIV-45-0-s002.docx]

**Supplementary Table S2 Polymorphisms, genotypes, allele frequencies and Hardy-Weinberg Equilibrium.**

| iCCA/pCCA | ID | Gene | *Genotype* | | | *Allele* | | | *HWE p-value* |
| --- | --- | --- | --- | --- | --- | --- | --- | --- | --- |
|  |  |  | *G* | n | % | *A* | n | % | *p* |
| iCCA | *rs1047768* | *ERCC5* | TT | 24 | 20.8 | T | 92 | 45.10 | 0.19 |
|  |  |  | TC | 44 | 50.5 | C | 112 | 54.90 |  |
|  |  |  | CC | 34 | 30.7 |  |  |  |  |
|  | *rs1130409* | *APEX1* | TT | 31 | 27.7 | T | 121 | 54.0 | *0.52* |
|  |  |  | TG | 59 | 52.7 | G | 103 | 46.0 |  |
|  |  |  | *GG* | 22 | 19.6 |  |  |  |  |
|  | *rs1805414* | *PARP1* | *AA* | 47 | 42.0 | A | 149 | 66.5 | *0.28* |
|  |  |  | *AG* | 55 | 49.1 | G | 75 | 33.5 |  |
|  |  |  | *GG* | 10 | 8.9 |  |  |  |  |
|  | *rs2228001* | *XPC* | *GG* | 19 | 17.0 | G | 90 | 40.2 | *0.72* |
|  |  |  | *GT* | 52 | 46.4 | T | 134 | 59.8 |  |
|  |  |  | *TT* | 41 | 36.6 |  |  |  |  |
|  | *rs873601* | *ERCC5* | *GG* | 11 | 9.8 | G | 71 | 32.0 | *0.88* |
|  |  |  | *GA* | 49 | 44.1 | A | 151 | 68.0 |  |
|  |  |  | *AA* | 51 | 45.9 |  |  |  |  |
| pCCA | *rs1047768* | *ERCC5* | TT | 26 | 24.5 | T | 113 | 44.84 | *0.14* |
|  |  |  | TC | 45 | 42.4 | C | 139 | 55.16 |  |
|  |  |  | CC | 35 | 33.0 |  |  |  |  |
|  | *rs1130409* | *APEX1* | TT | *39* | *33.3* | *T* | *129* | *56.1* | *0.76* |
|  |  |  | TG | *55* | *47.0* | *G* | *101* | *43.9* |  |
|  |  |  | *GG* | *23* | *19.7* |  |  |  |  |
|  | *rs1805414* | *PARP1* | *AA* | *59* | *50.4* | *A* | *162* | *69.2* | 0.20 |
|  |  |  | *AG* | *44* | *37.6* | *G* | *72* | *30.8* |  |
|  |  |  | *GG* | *14* | *12.0* |  |  |  |  |
|  | *rs2228001* | *XPC* | *GG* | *21* | *17.9* | *G* | *103* | *44.0* | *0.53* |
|  |  |  | *GT* | *61* | *52.1* | *T* | *131* | *56.0* |  |
|  |  |  | *TT* | *35* | *29.9* |  |  |  |  |
|  | *rs873601* | *ERCC5* | *GG* | *3* | *2.6* | *G* | *43* | *18.4* | *0.56* |
|  |  |  | *GA* | *37* | *31.6* | *A* | *191* | *81.6* |  |
|  |  |  | *AA* | *77* | *65.8* |  |  |  |  |

APEX1, apurinic/apyrimidinic endodeoxyribonuclease 1; ERCC5, ERCC excision repair 5; iCCA, intrahepatic cholangiocarcinoma; PARP1, poly(ADP-ribose) polymerase 1; pCCA, perihilar cholangiocarcinoma; XPC, Xeroderma pigmentosum complementation group C.
