# Supplementary material for: The Prognostic Value of the XPC rs2228001 Single Nucleotide Polymorphism in Cholangiocarcinoma
Source: Liver Int. 2025 Aug 20;45(9):e70292. doi: 10.1111/liv.70292 (PMC12366541; doi:10.1111/liv.70292)
Supplement: Supplementary file 6 — Table S5: Single nucleotide polymorphism frequencies and associations with recurrence‐free survival, cancer‐specific survival and overall survival in intrahepatic cholangiocarcinoma without adjuvant therapy. [file LIV-45-0-s005.docx]

**Supplementary table S5 Single nucleotide polymorphism frequencies and associations with recurrence-free survival, cancer-specific survival and overall survival in intrahepatic cholangiocarcinoma without adjuvant therapy**

| **SNP** | **N (%)** | **Recurrence-free survival** | | | |  | **Cancer-specific survival** | | | |  | **Overall survival** | | | |
| --- | --- | --- | --- | --- | --- | --- | --- | --- | --- | --- | --- | --- | --- | --- | --- |
|  |  | **Median**  **(95% CI)** | **p value*** | **HR (95% CI)** | **p value^#^** |  | **Median**  **(95% CI)** | **p value*** | **HR (95% CI)** | **p value^#^** |  | **Median**  **(95% CI)** | **p value*** | **HR (95% CI)** | **p value^#^** |
| **Recessive model** | | | | | | | | | | | | | | | |
| **rs1047768** |  |  | 0.547 |  |  |  |  | 0.938 |  |  |  |  | 0.460 |  |  |
| TT/TC | 41(59.4) | 11(3.2-18.8) |  | 1 |  |  | 31(8.5-52.5) |  | 1 |  |  | 22(6.5-37.5) |  | 1 |  |
| CC | 21(30.4) | 20(0-47.1) |  | 1.224(0.626-2.396) | 0.555 |  | 39(18.7-59.3) |  | 0.974(0.493-1.923)- | 0.938 |  | 39(13.7-64.8) |  | 0.790(0.420-1.486) | 0.465 |
| **rs1130409** |  |  | **0.021** |  |  |  |  | 0.388 |  |  |  |  | 0.088 |  |  |
| TT/TG | 58(84.1) | 17(7.1-26.9) |  | 1 |  |  | 31(12.3-49.7) |  | 1 |  |  | 25(10.6-39.4) |  | 1 |  |
| GG | 11(15.9) | 5(1.8-8.4) |  | 2.737(1.089-6.878) | 0.032 |  | 25(0-65.1) |  | 1.459(0.612-3.481) | 0.394 |  | 13(0-26.6) |  | 1.849(0.896-3.819) | 0..096 |
| **rs1805414** |  |  | 0.689 |  |  |  |  | 0.182 |  |  |  |  | **0.023** |  |  |
| AA/AG | 63(91.3) | 13(2.9-23.0) |  | 1 |  |  | 36(19.6-52.4) |  | 1 |  |  | 25(10.9-39.1) |  | 1 |  |
| GG | 6(8.7) | 8(1.8-14.2) |  | 1.264(0.387-4.127) | 0.698 |  | 11(6.0-16.0) |  | 2.204(0.661-7.353) | 0.198 |  | 5(0-10.6) |  | 1.573(0.537-4.609) | 0.409 |
| **rs2228001** |  |  | 0.205 |  |  |  |  | 0.322 |  |  |  |  | 0.326 |  |  |
| GG/GT | 41(59.4) | 8(0-17.4) |  | 1 |  |  | 25(5.4-44.6) |  | 1 |  |  | 16(9.9-22.1) |  | 1 |  |
| TT | 28(40.6) | 19(0-44.8) |  | 0.676(0.362-1.262) | 0.219 |  | 46(30.6-61.4) |  | 0.730(0.389-1.371) | 0.328 |  | 41(7.4-74.6) |  | 0.756(0.430-1.330) | 0.332 |
| **rs873601** |  |  | 0.197 |  |  |  |  | 0.062 |  |  |  |  | 0.159 |  |  |
| GG/GA | 33(47.8) | 15(0-33.3) |  | 1 |  |  | -- |  | 1 |  |  | 22(1.3-42.7) |  | 1 |  |
| AA | 36(52.2) | 8(4.4-11.6) |  | 1.481(0.802-2.736) | 0.210 |  | 25(12.7-37.3) |  | 1.827(0.956-3.492) | 0.068 |  | 25(17.0-33.0) |  | 1.490(0.847-2.621) | 0.166 |
| **Co-dominant model** | |  |  |  |  |  |  |  |  |  |  |  |  |  |  |
| **rs1047768** |  |  | 0.395 |  |  |  |  | 0.400 |  |  |  |  | 0.222 |  |  |
| TT | 14(20.3) | 26(0-56.2) |  | 1 |  |  | -- |  | 1 |  |  | 18.0(0-85.7) |  | 1 |  |
| TC | 27(39.1) | 8(2.5) |  | 1.637(0.704-3.807) | 0.252 |  | 27(11.2-42.8) |  | 1.879(0.735-4.805) | 0.188 |  | 22(11.0-32.9) |  | 1.807(0.826-3.953) | 0.138 |
| CC | 21(30.4) | 20(0-47.1) |  | 1.109(0.459-2.678) | 0.818 |  | 39(18.7-59.3) |  | 1.487(0.564-3.922) | 0.423 |  | 39(13.7-64.3) |  | 1.169(0.501-2.730) | 0.718 |
| **rs1130409** |  |  | 0.069 |  |  |  |  | 0.502 |  |  |  |  | 0.198 |  |  |
| TT | 25(36.2) | 19(0-58.6) |  | 1 |  |  | 27(2.0-52.0) |  | 1 |  |  | 21(0-43.8) |  | 1 |  |
| TG | 33(47.8) | 15(3.6-26.4) |  | 0.939(0.483-1.826) | 0.854 |  | 31(13.8-48.2) |  | 0.761(0.391-1.482) | 0.422 |  | 38(17.5-58.5) |  | 0.836(0.460-1.521) | 0.558 |
| GG | 11(15.9) | 5(1.6-8.4) |  | 2.636(0.967-7.184) | 0.058 |  | 39(3.6-74.4) |  | 1.247(0.486-3.197) | 0.646 |  | 13(0-26.6) |  | 1.666(0.749-3.705) | 0.211 |
| **rs1805414** |  |  | 0.642 |  |  |  |  | 0.215 |  |  |  |  | **0.032** |  |  |
| AA | 28(40.6) | 23(10.3-35.7) |  | 1 |  |  | 39(31.9-46.0) |  | 1 |  |  | 36(24.8-47.2) |  | 1 |  |
| AG | 35(50.7) | 8(3.0-12.9) |  | 1.316(0.692-2.501) | 0.402 |  | 22(13.8-30.2) |  | 1.465(0.762-2.815) | 0.252 |  | 17(6.7-27.2) |  | 1.478(0.826-2.644) | 0.188 |
| GG | 6(8.7) | 8(1.8-14.2) |  | 1.472(0.425-5.096) | 0.504 |  | 11(6.0-15.9) |  | 2.747(0.771-9.786) | 0.119 |  | 5(0-10.6) |  | 3.578(1.288-9.943) | 0.014 |
| **rs2228001** |  |  | 0.308 |  |  |  |  | 0.423 |  |  |  |  | 0.617 |  |  |
| GG | 10(14.5) | 15(0.9-29.0) |  | 1 |  |  | -- |  | 1 |  |  | 17(9.9-24.1) |  | 1 |  |
| GT | 31(449) | 7(2.1-11.8) |  | 1.471(0.554-3.906) | 0.439 |  | 25(9.6-40.4) |  | 1.543(0.528-4.515) | 0.428 |  | 13(0-30.4) |  | 0.993(0.426-2.318) | 0.988 |
| TT | 28(40.6) | 19(0-44.4) |  | 0.912(0.334-2.492) | 0.857 |  | 46(30.6-61.4) |  | 1.037(0.345-3.115) | 0.948 |  | 41(7.4-74.6) |  | 0.753(0.317-1.788) | 0.520 |
| **rs873601** |  |  | 0.247 |  |  |  |  | 0.159 |  |  |  |  | 0.316 |  |  |
| GG | 5(7.2) | --- |  | 1 |  |  | -- |  | 1 |  |  | 17(0-40.6) |  | 1 |  |
| GA | 28(40.6) | 13(4.9-21.0) |  | 3.076(0.409-23.150) | 0.275 |  | -- |  | 1.718(0.244-13.159) | 0.603 |  | 36(5.3-66.7) |  | 0.707(0.236-2.123) | 0.537 |
| AA | 36(52.2) | 8(4.4-11.5) |  | 4.080(0.551-30.193) | 0.168 |  | 25(12.7-37.3) |  | 2.986(0.405-22.030) | 0.283 |  | 25(16.9-33.0) |  | 1.107(0.376-3.259) | 0.854 |
| **Dominant model** | |  |  |  |  |  |  |  |  |  |  |  |  |  |  |
| **rs1047768** |  |  | 0.436 |  |  |  |  | 0.240 |  |  |  |  | 0.279 |  |  |
| TT | 14(20.3) | 26(0-56.2) |  | 1 |  |  | 31(15.2-46.8) |  | 1 |  |  | 25(14.1-35.9) |  | 1 |  |
| TC/CC | 48(69.6) | 13(2.3-23.6) |  | 1.355(0.621-2.961) | 0.445 |  | -- |  | 1.683(0.697-4.063) | 0.247 |  | 18(0-85.7) |  | 1.498(0.714-3.144) | 0.285 |
| **rs1130409** |  |  | 0.796 |  |  |  |  | 0.582 |  |  |  |  | 0.897 |  |  |
| TT | 25(36.2) | 19(0-58.6) |  | 1 |  |  | 27(1.978-52.0) |  | 1 |  |  | 21(0-43.8) |  | 1 |  |
| GT/GG | 44(63.8) | 11(6.9-15.0) |  | 1.085(0.576-2.045) | 0.801 |  | 38(11.3-64.7) |  | 0.839(0.447-1.575) | 0.585 |  | 25(2.7-47.3) |  | 0.964(0.549-1.692) | 0.898 |
| **rs1805414** |  |  | 0.357 |  |  |  |  | 0.177 |  |  |  |  | 0.095 |  |  |
| AA | 28(40.6) | 23(10.3-35.7) |  | 1 |  |  | 39(31.9-46.0) |  | 1 |  |  | 13(6.8-19.2) |  | 1 |  |
| AG/GG | 41(59.4) | 8(4.1-11.9) |  | 1.332(0.712-2.493) | 0.370 |  | 21(10.5-31.5) |  | 1.543(0.814-2.924) | 0.183 |  | 36(24.8-47.2) |  | 1.606(0.912-2.828) | 0.101 |
| **rs2228001** |  |  | 0.746 |  |  |  |  | 0.642 |  |  |  |  | 0.724 |  |  |
| GG | 10(14.5) | 15(0.9-29.1) |  | 1 |  |  | -- |  | 1 |  |  | 17(9.9-24.1) |  | 1 |  |
| GT/TT | 59(85.5) | 13(1.1-24.9) |  | 1.162(0.456-2.961) | 0.752 |  | 31(14.7-47.3) |  | 1.275(0.453-3.590) | 0.646 |  | 25(12.6-37.3) |  | 0.867(0.389-1.934) | 0.727 |
| **rs873601** |  |  | 0.166 |  |  |  |  | 0.367 |  |  |  |  | 0.828 |  |  |
| GG | 5(7.2) | -- |  | 1 |  |  | -- |  | 1 |  |  | 17(0-40.6) |  | 1 |  |
| GA/AA | 64(92.8) | 11(5.9-16.1) |  | 3.596(0.494-26.173) | 0.206 |  | 31(11.9-50.0) |  | 2.405(0.330-17.535) | 0.387 |  | 25(10.9-39.1) |  | 0.892(0.316-2.522) | 0.830 |

*，Kaplan–Meier survival analysis；#，univariate Cox regression analyses
