# Supplementary material for: The Prognostic Value of the XPC rs2228001 Single Nucleotide Polymorphism in Cholangiocarcinoma
Source: Liver Int. 2025 Aug 20;45(9):e70292. doi: 10.1111/liv.70292 (PMC12366541; doi:10.1111/liv.70292)
Supplement: Supplementary file 7 — Table S6: Single nucleotide polymorphism frequencies and associations with recurrence‐free survival, cancer‐specific survival and overall survival in intrahepatic cholangiocarcinoma with adjuvant therapy. [file LIV-45-0-s006.docx]

**Supplementary table S6 Single nucleotide polymorphism frequencies and associations with recurrence-free survival, cancer-specific survival and overall survival in intrahepatic cholangiocarcinoma with adjuvant therapy**

| **SNP** | **N (%)** | **Recurrence-free survival** | | | |  | **Cancer-specific survival** | | | |  | **Overall survival** | | | |
| --- | --- | --- | --- | --- | --- | --- | --- | --- | --- | --- | --- | --- | --- | --- | --- |
|  |  | **Median**  **(95% CI)** | **p value*** | **HR (95% CI)** | **p value^#^** |  | **Median**  **(95% CI)** | **p value*** | **HR (95% CI)** | **p value^#^** |  | **Median**  **(95% CI)** | **p value*** | **HR (95% CI)** | **p value^#^** |
| **Recessive model** | | | | | | | | | | | | | | | |
| **rs1047768** |  |  | 0.883 |  |  |  |  | 0.776 |  |  |  |  | 0.776 |  |  |
| TT/TC | 26(61.9) | 13(8.3-17.7) |  | 1 |  |  | 28(13.1-42.9) |  | 1 |  |  | 28(13.1-42.9) |  | 1 |  |
| CC | 13(31.0) | 9(4.3-13.7) |  | 1.057(0.498-2.241) | 0.886 |  | 27(20.4-33.6) |  | 0.886(0.383-2.050) | 0.777 |  | 28(20.8-35.2) |  | 0.886(0.383-2.050) | 0.777 |
| **rs1130409** |  |  | 0.338 |  |  |  |  | 0.136 |  |  |  |  | 0.136 |  |  |
| TT/TG | 31(73.9) | 13(4.8-21.2) |  | 1 |  |  | 29(11.8-46.2) |  | 1 |  |  | 29(11.8-46.2) |  | 1 |  |
| GG | 11(26.2) | 9(4.0-14.0) |  | 1.448(0.660-3.178) | 0.356 |  | 22(7.9-36.0) |  | 1.827(0.812-4.114) | 0.145 |  | 22(8.0-36.0) |  | 1.827(0.812-4.114) | 0.145 |
| **rs1805414** |  |  | 0.989 |  |  |  |  | 0.401 |  |  |  |  | 0.401 |  |  |
| AA/AG | 38(90.5) | 10.0(5.5-14.5) |  | 1 |  |  | 28(20.6-35.4) |  | 1 |  |  | 28(20.6-35.4) |  | 1 |  |
| GG | 4(9.5) | 13(7.0-18.9) |  | 0.992(0.298-3.295) | 0.989 |  | 20(2.4-37.6) |  | 1.573(0.537-4.609) | 0.409 |  | 20(2.4-37.6) |  | 2.851(1.097-7.408) | **0.032** |
| **rs2228001** |  |  | 0.109 |  |  |  |  | 0.056 |  |  |  |  | 0.056 |  |  |
| GG/GT | 29(69.0) | 10(3.0-17.0) |  | 1 |  |  | 46(30.6-61.4) |  | 1 |  |  | 25(16.2-33.8) |  | 1 |  |
| TT | 13(31.0) | 22(2.9-41.1) |  | 0.545(0.251-1.183) | 0.125 |  | 28(21.7-34.3) |  | 0.398(0.149-1.064) | 0.066 |  | -- |  | 0.398(0.149-1.064) | 0.066 |
| **rs873601** |  |  | 0.146 |  |  |  |  | 0.850 |  |  |  |  | 0.850 |  |  |
| GG/GA | 31(36.5) | 13(5.9-20.1) |  | 1 |  |  | 28(19.3-36.7) |  | 1 |  |  | 28(19.3-36.7) |  | 1 |  |
| AA | 54(63.5) | 10(3.7-16.3) |  | 1.633(0.821-3.247) | 0.162 |  | 27(11.9-42.1) |  | 1.075(0.503-2.300) | 0.851 |  | 27(11.9-42.1) |  | 1.075(0.503-2.300) | 0.851 |
| **Co-dominant model** | |  |  |  |  |  |  |  |  |  |  |  |  |  |  |
| **rs1047768** |  |  | 0.733 |  |  |  |  | 0.728 |  |  |  |  | 0.728 |  |  |
| TT | 10(23.8) | 7(6.2-7.8) |  | 1 |  |  | 20(17.0-23.0) |  | 1 |  |  | 20(16.9-23.0) |  | 1 |  |
| TC | 16(38.1) | 15(10.1-19.9) |  | 0.723(0.307-1.701) | 0.457 |  | 32(15.2-48.8) |  | 0.706(0.274-1.818) | 0.470 |  | 32(15.2-48.8) |  | 0.706(0.274-1.818) | 0.470 |
| CC | 13(31.0) | 9(4.3-13.7) |  | 0.771(0.311-1.911) | 0.574 |  | 20(17.0-23.0) |  | 0.723(0.270-1.934) | 0.518 |  | 27(20.4-33.6) |  | 0.723(0.270-1.934) | 0.518 |
| **rs1130409** |  |  | 0.470 |  |  |  |  | 0.240 |  |  |  |  | 0.240 |  |  |
| TT | 5(11.9) | 20(11.4-28.6) |  | 1 |  |  | -- |  | 1 |  |  | -- |  | 1 |  |
| TG | 26(61.9) | 10(2.5-17.5) |  | 1.524(0.522-4.450) | 0.441 |  | 27(19.5-34.5) |  | 1.879(0.431-8.196) | 0.401 |  | 27(19.5-34.5) |  | 1.879(0.431-8.196) | 0.401 |
| GG | 11(26.2) | 9(4.0-14.0) |  | 2.045(0.615-6.808) | 0.243 |  | 22(8.0-36.0) |  | 3.121(0.672-14.504) | 0.147 |  | 22(19.5-34.5) |  | 3.121(0.672-14.504) | 0.147 |
| **rs1805414** |  |  | 0.390 |  |  |  |  | 0.240 |  |  |  |  | 0.507 |  |  |
| AA | 19(45.2) | 16(3.2-28.8) |  | 1 |  |  | -- |  | 1 |  |  | -- |  | 1 |  |
| AG | 19(45.2) | 10(7.2-12.8) |  | 1.623(0.792-3.328) | 0.186 |  | 29(20.5-37.5) |  | 1.403(0.623-3.162) | 0.413 |  | 29(20.5-37.5) |  | 1.403(0.623-3.162) | 0.413 |
| GG | 4(9.5) | 13(7.0-18.9) |  | 1.280(0.359-4.569) | 0.703 |  | 20(2.4-37.6) |  | 1.889(0.584-6.111) | 0.288 |  | 20(2.4-37.6) |  | 1.889(0.584-6.111) | 0.288 |
| **rs2228001** |  |  | **0.007** |  |  |  |  | **0.004** |  |  |  |  | **0.004** |  |  |
| GG | 8(19.0) | 17(8.7-25.3) |  | 1 |  |  | 42(33.5-50.5) |  | 1 |  |  | 42(33.5-50.5) |  | 1 |  |
| GT | 21(50.0) | 7(6.1-7.8) |  | 3.379(1.212-9.421) | 0.020 |  | 20(16.6-23.4) |  | 3.806(1.242-11.663) | 0.019 |  | 20(16.6-23.4) |  | 3.806(1.242-11.663) | 0.019 |
| TT | 13(31.0) | 22(2.9-41.0) |  | 1.175(0.393-3.511) | 0.773 |  | -- |  | 0.970(0.257-3.664) | 0.964 |  | -- |  | 0.970(0.257-3.664) | 0.964 |
| **rs873601** |  |  | 0.345 |  |  |  |  | 0.553 |  |  |  |  | 0.553 |  |  |
| GG | 6(14.3) | 9(5.3-12.6) |  | 1 |  |  | 84(50.4-117.6) |  | 1 |  |  | 19(0-39.4) |  | 1 |  |
| GA | 20(47.6) | 13(4.2-21.7) |  | 0.941(0.311-2.848) | 0.914 |  | 33(0-67.5) |  | 0.566(0.198-1.618) | 0.288 |  | 28(15.5-40.4) |  | 0.566(0.198-1.618) | 0.288 |
| AA | 15(35.7) | 10(3.6-16.3) |  | 1.555(0.508-4.762) | 0.439 |  | 65(10.2-119.8) |  | 0.697(0.240-2.023) | 0.506 |  | 27(11.9-42.1) |  | 0.697(0.240-2.023) | 0.506 |
| **Dominant model** | |  |  |  |  |  |  |  |  |  |  |  |  |  |  |
| **rs1047768** |  |  | 0.439 |  |  |  |  | 0.426 |  |  |  |  | 0.246 |  |  |
| TT | 10(23.8) | 7(6.2-7.7) |  | 1 |  |  | 20(16.9-23.0) |  | 1 |  |  | 20(16.9-23.0) |  | 1 |  |
| TC/CC | 29(69.0) | 13(6.7-19.2) |  | 0.743(0.342-1.615) | 0.453 |  | 30(21.6-38.4) |  | 0.713(0.307-1.656) | 0.432 |  | 30(21.6-38.4) |  | 0.713(0.307-1.656) | 0.432 |
| **rs1130409** |  |  | 0.339 |  |  |  |  | 0.268 |  |  |  |  |  |  |  |
| TT | 5(11.9) | 20(11.4-28.6) |  | 1 |  |  | -- |  | 1 |  |  | -- |  | 1 |  |
| GT/GG | 37(88.1) | 10(5.1-14.9) |  | 1.638(0.574-4.673) | 0.357 |  | 39(18.7-35.3) |  | 2.197(0.519-9.291) | 0.285 |  | 27 (18.7-35.3) |  | 2.197(0.519-9.291) | 0.285 |
| **rs1805414** |  |  | 0.192 |  |  |  |  | 0.308 |  |  |  |  | 0.308 |  |  |
| AA | 19(45.2) | 16(3.2-28.8) |  | 1 |  |  | 28(20.2-35.8) |  | 1 |  |  | -- |  | 1 |  |
| AG/GG | 23(54.8) | 10(6.9-13.0) |  | 1.565(0.779-3.145) | 0.209 |  | -- |  | 1.487(0.686-3.225) | 0.315 |  | 28(20.2-35.8) |  | 1.487(0.686-3.225) | 0.315 |
| **rs2228001** |  |  | 0.128 |  |  |  |  | 0.120 |  |  |  |  | 0.120 |  |  |
| GG | 8(19.0) | 17(8.7-25.3) |  | 1 |  |  | 42(33.5-50.5) |  | 1 |  |  | 42(33.5-50.5) |  | 1 |  |
| GT/TT | 34(81.0) | 9(4.7-13.3) |  | 2.028(0.780-5.276) | 0.147 |  | 24(14.0-34.0) |  | 2.295(0.777-6.779) | 0.133 |  | 24(14.0-34.0) |  | 2.295(0.777-6.779) | 0.133 |
| **rs873601** |  |  | 0.776 |  |  |  |  | 0.330 |  |  |  |  | 0.330 |  |  |
| GG | 6(14.3) | 9(5.4-12.6) |  | 1 |  |  | 19(0-39.4) |  | 1 |  |  | 19(0-39.4) |  | 1 |  |
| GA/AA | 35(83.3) | 12(7.4-16.6) |  | 1.159(0.404-3.323) | 0.783 |  | 28(18.9-37.0) |  | 0.622(0.234-1.648) | 0.339 |  | 28(18.9-37.0) |  | 0.622(0.234-1.648) | 0.339 |

*，Kaplan–Meier survival analysis；#，univariate Cox regression analyses
