# Supplementary material for: The Prognostic Value of the XPC rs2228001 Single Nucleotide Polymorphism in Cholangiocarcinoma
Source: Liver Int. 2025 Aug 20;45(9):e70292. doi: 10.1111/liv.70292 (PMC12366541; doi:10.1111/liv.70292)
Supplement: Supplementary file 8 — Table S7: Single nucleotide polymorphism frequencies and associations with recurrence‐free survival, cancer‐specific survival and overall survival in perihilar cholangiocarcinoma without adjuvant therapy. [file LIV-45-0-s001.docx]

**Supplementary table S7 Single nucleotide polymorphism frequencies and associations with recurrence-free survival, cancer-specific survival, and overall survival in perihilar cholangiocarcinoma without adjuvant therapy**

**.**

| **SNP** | **N (%)** | **Recurrence-free survival** | | | |  | **Cancer-specific survival** | | | |  | **Overall Survival** | | | |
| --- | --- | --- | --- | --- | --- | --- | --- | --- | --- | --- | --- | --- | --- | --- | --- |
|  |  | **Median**  **(95% CI)** | **p value*** | **HR (95% CI)** | **p value^#^** |  | **Median**  **(95% CI)** | **P value*** | **HR (95% CI)** | **p value^#^** |  | **Median**  **(95% CI)** | **p value*** | **HR (95% CI)** | **p value^#^** |
| **Recessive model** | |  |  |  |  |  |  |  |  |  |  |  |  |  |  |
| **rs1047768** |  |  | 0.606 |  |  |  |  | 0.543 |  |  |  |  | 0.527 |  |  |
| TT/TC | 54(63.5) | 24.8(0-93.6) |  | 1 |  |  | 50(11.9-88.1) |  | 1 |  |  | 30(19.0-40.9) |  | 1 |  |
| CC | 22(25.9) | 32.3(14.7-141.3) |  | 1.210(0.580-2.524) | 0.611 |  | 65(30.3-99.8) |  | 0.799(0.385-1.657) | 0.546 |  | 54(9.6-98.4) |  | 0.830(0.463-1.488) | 0.531 |
| **rs1130409** |  |  | 0.424 |  |  |  |  | 0.200 |  |  |  |  | 0.494 |  |  |
| TT/TG | 66(77.6) | 69(17.8-120.2) |  | 1 |  |  | 76(34.4-117.6) |  | 1 |  |  | 33(10.6-55.4) |  | 1 |  |
| GG | 19(22.4) | 45(17.4-72.6) |  | 1.310(0.670-2.563) | 0.430 |  | 45(11.1-78.7) |  | 1.509(0.799-2.852) | 0.205 |  | 41(21.8-60.2) |  | 1.212(0.694-2.117) | 0.499 |
| **rs1805414** |  |  | **0.087** |  |  |  |  | 0.378 |  |  |  |  | 0.189 |  |  |
| AA/AG | 76(89.4) | 67(30.2-103.8) |  | 1 |  |  | 63(29.8-96.2) |  | 1 |  |  | 41(17.5-64.5) |  | 1 |  |
| GG | 9(10.6) | 24(16.4-31.6) |  | 2.103(0.873-5.068) | 0.097 |  | 54(21.0-87.0) |  | 1.472(0.617-3.515) | 0.384 |  | 32(26.2-37.8) |  | 1.573(0.537-4.609) | 0.409 |
| **rs2228001** |  |  | 0.324 |  |  |  |  | **0.085** |  |  |  |  | 0.212 |  |  |
| GG/GT | 62(72.9) | 42(18.7-65.3) |  | 1 |  |  | 49(22.4-75.6) |  | 1 |  |  | 32(18.3-48.7) |  | 1 |  |
| TT | 23(27.1) | 78(52.9-103.1) |  | 0.702(0.344-1.432) | 0.331 |  | -- |  | 0.517(0.240-1.113) | 0.092 |  | 63(18.8-107.2) |  | 0.695(0.389-1.241) | 0.218 |
| **rs873601** |  |  | 0.025 |  |  |  |  | 0.342 |  |  |  |  | 0.689 |  |  |
| GG/GA | 31(36.5) | 24(0-63.4) |  | 1 |  |  | 50(7.5-92.5) |  | 1 |  |  | 49(20.8-77.2) |  | 1 |  |
| AA | 54(63.5) | --- |  | 0.509(0.278-0.934) | 0.029 |  | 65(10.2-119.8) |  | 0.751(0.415-1.361) | 0.345 |  | 32(18.2-45.8) |  | 1.109(0.665-1.850) | 0.692 |
| **Co-dominant model** | |  |  |  |  |  |  |  |  |  |  |  |  |  |  |
| **rs1047768** |  |  | 0.806 |  |  |  |  | 0.292 |  |  |  |  | 0.458 |  |  |
| TT | 24(28.2) | 83(0-174.7) |  | 1 |  |  | 25(12.5.37.5-) |  | 1 |  |  | 19(6.4-31.6) |  | 1 |  |
| TC | 30(35.3) | 45(0-93.4) |  | 0.855(0.388-1.886) | 0.698 |  | -- |  | 0.586(0.275-1.252) | 0.168 |  | 38(18.6-57.4) |  | 0.720(0.386-1.342) | 0.301 |
| CC | 22(25.9) | 78(14.7-141.3) |  | 0.754(0.320-1.780) | 0.520 |  | 76(35.8-116.2) |  | 0.595(0.263-1.348) | 0.214 |  | 54(9.6-98.4) |  | 0.689(0.352-1.345) | 0.275 |
| **rs1130409** |  |  | 0.578 |  |  |  |  | 0.289 |  |  |  |  | 0.292 |  |  |
| TT | 27(31.8) | 83(15.2-150.8) |  | 1 |  |  | -- |  | 1 |  |  | 56(22.2-89.8) |  | 1 |  |
| TG | 39(45.9) | 67(4.8-129.2) |  | 1.298(0.621-2.716) | 0.488 |  | 27(19.5-34.5) |  | 1.445(0.681-3.066) | 0.337 |  | 30(23.2-36.8) |  | 1.552(0.847-2.844) | 0.155 |
| GG | 19(22.4) | 45(17.3-72.6) |  | 1.521(0.680-3.400) | 0.307 |  | 22(7.9-36.0) |  | 1.879(0.848-4.165) | 0.120 |  | 42(21.8-60.2) |  | 1.577(0.798-3.118) | 0.190 |
| **rs1805414** |  |  | 0.211 |  |  |  |  | 0.530 |  |  |  |  | 0.353 |  |  |
| AA | 45(52.9) | 67.0(21.0-112.9) |  | 1 |  |  | -- |  | 1 |  |  | 41(15.1-66.9) |  | 1 |  |
| AG | 31(36.5) | 69(15.3-122.8) |  | 1.163(0.602-2.245) | 0.654 |  | 54(10.6-97.4) |  | 1.261(0.667-2.386) | 0.475 |  | 45(13.5-76.5) |  | 1.180(0.693-2.010) | 0.542 |
| GG | 9(10.6) | 24(16.4-31.6) |  | 2.241(0.888-5.656) | 0.088 |  | 33(28.6-37.4) |  | 1.630(0.650-4.091) | 0.298 |  | 32(26.2-37.8) |  | 1.716(0.809-3.641) | 0.159 |
| **rs2228001** |  |  | 0.392 |  |  |  |  | 0.196 |  |  |  |  | 0.166 |  |  |
| GG | 15(17.6) | 69(25.3-112.6) |  | 1 |  |  | 65(7.9-122.1) |  | 1 |  |  | 51(14.8-87.2) |  | 1 |  |
| GT | 47(55.3) | 35(6.4-63.5) |  | 1.469(0.632-3.413) | 0.371 |  | 49(38.4-69.6) |  | 1.212(0.567-2.589) | 0.620 |  | 31(26.2-35.8) |  | 1.611(0.800-3.245) | 0.181 |
| TT | 23(27.1) | 78(52.9-103.0) |  | 0.933(0.355-2.454) | 0.888 |  | -- |  | 0.594(0.229-1.539) | 0.283 |  | 63(18.8-107.2) |  | 0.992(0.445-2.209) | 0.984 |
| **rs873601** |  |  | 0.076 |  |  |  |  | 0.627 |  |  |  |  | 0.850 |  |  |
| GG | 3(3.5) | 55(50.1-59.8) |  | 1 |  |  | 84(50.4-117.6) |  | 1 |  |  | 84(50.4-117.6) |  | 1 |  |
| GA | 28(32.9) | 24(12.1-35.8) |  | 1.196(0.352-4.065) | 0.774 |  | 33(0-39.4) |  | 1.102(0.321-3.787) | 0.877 |  | 33(66.2-59.8) |  | 1.293(0.382-4.374) | 0.679 |
| AA | 54(63.5) | -- |  | 0.593(0.175-2.006) | 0.401 |  | 65(10.2-119.8) |  | 0.816(0.244-2.724) | 0.741 |  | 32(18.2-45.8) |  | 1.382(0.426-4.484) | 0.590 |
| **Dominant model** | |  |  |  |  |  |  |  |  |  |  |  |  |  |  |
| **rs1047768** |  |  | 0.558 |  |  |  |  | 0.117 |  |  |  |  | 0.214 |  |  |
| TT | 24(28.2) | 83(0-174.6) |  | 1 |  |  | 76(39.4-112.6) |  | 1 |  |  | 19(6.4-31.6) |  | 1 |  |
| TC/CC | 52(61.2) | 67(25.7-108.2) |  | 0.810(0.396-1.657) | 0.564 |  | 25(12.5-37.5) |  | 0.590(0.302-1.153) | 0.123 |  | 49(25.1-72.9) |  | 0.706(0.405-1.232) | 0.220 |
| **rs1130409** |  |  | 0.344 |  |  |  |  | 0.175 |  |  |  |  | 0.117 |  |  |
| TT | 27(31.8) | 83(15.2-150.8) |  | 1 |  |  | -- |  | 1 |  |  | 56(22.2-89.8) |  | 1 |  |
| GT/GG | 58(68.2) | 45(17.2-72.8) |  | 1.380(0.702-2.714) | 0.350 |  | 45(21.6-68.3) |  | 1.604(0.804-3.202) | 0.180 |  | 32(21.6-42.4) |  | 1.561(0.887-2.748) | 0.123 |
| **rs1805414** |  |  | 0.343 |  |  |  |  | 0.335 |  |  |  |  | 0.312 |  |  |
| AA | 45(52.9) | 67(21.0-113.0) |  | 1 |  |  | -- |  | 1 |  |  | 41(15.1-66.9) |  | 1 |  |
| AG/GG | 40(47.1) | 37(0-77.6) |  | 1.337(0.729-2.451) | 0.348 |  | 49(0-98.3) |  | 1.337(0.738-2.421) | 0.338 |  | 33(17.2-48.8) |  | 1.286(0.786-2.104) | 0.317 |
| **rs2228001** |  |  | 0.577 |  |  |  |  | 0.944 |  |  |  |  | 0.360 |  |  |
| GG | 15(17.6) | 69(25.4-112.6) |  | 1 |  |  | 65(7.8-122.1) |  | 1 |  |  | 51(14.8-87.2) |  | 1 |  |
| GT/TT | 70(82.4) | 52(14.2-89.8) |  | 1.258(0.557-2.839) | 0.581 |  | 54(12.7-95.3) |  | 0.974(0.467-2.031) | 0.944 |  | 32(15.3-48.7) |  | 1.366(0.694-2.689) | 0.366 |
| **rs873601** |  |  | 0.685 |  |  |  |  | 0.879 |  |  |  |  | 0.608 |  |  |
| GG | 3(3.5) | 55(50.2-59.8) |  | 1 |  |  | 19(0-39.4) |  | 1 |  |  | 84(50.4-117.6) |  | 1 |  |
| GA/AA | 82(96.5) | 45(5.7-84.3) |  | 0.785(0.241-2.554) | 0.688 |  | 84(50.4-117.6) |  | 0.913(0.281-2.967) | 0.880 |  | 32(19.7-44.3) |  | 1.352(0.422-4.329) | 0.612 |

*，Kaplan–Meier survival analysis；#，univariate Cox regression analyses
