# Supplementary material for: The Prognostic Value of the XPC rs2228001 Single Nucleotide Polymorphism in Cholangiocarcinoma
Source: Liver Int. 2025 Aug 20;45(9):e70292. doi: 10.1111/liv.70292 (PMC12366541; doi:10.1111/liv.70292)
Supplement: Supplementary file 9 — Table S8: Single nucleotide polymorphism frequencies and associations with recurrence‐free survival, cancer‐specific survival and overall survival in perihilar cholangiocarcinoma with adjuvant therapy. [file LIV-45-0-s009.docx]

**Supplementary table S8 Single nucleotide polymorphism frequencies and associations with recurrence-free survival, cancer-specific survival, and overall survival in perihilar cholangiocarcinoma with adjuvant therapy.**

| **SNP** | **N (%)** | **Recurrence-free survival** | | | |  | **Cancer-specific survival** | | | |  | **Overall Survival** | | | |
| --- | --- | --- | --- | --- | --- | --- | --- | --- | --- | --- | --- | --- | --- | --- | --- |
|  |  | **Median**  **(95% CI)** | **p value*** | **HR (95% CI)** | **p value^#^** |  | **Median**  **(95% CI)** | **P value*** | **HR (95% CI)** | **p value^#^** |  | **Median**  **(95% CI)** | **p value*** | **HR (95% CI)** | **p value^#^** |
| **Recessive model** | |  |  |  |  |  |  |  |  |  |  |  |  |  |  |
| **rs1047768** |  |  | 0.909 |  |  |  |  | 0.795 |  |  |  |  | 0.490 |  |  |
| TT/TC | 17(54.8) | 16(10.1-21.9) |  | 1 |  |  | 33(0-66.9) |  | 1 |  |  | 33(2.7-63.3) |  | 1 |  |
| CC | 12(38.7) | 22(1.5-42.5) |  | 0.949(0.381-2.366) | 0.910 |  | 39(16.4-61.6) |  | 0.872(0.308-2.467) | 0.796 |  | 39(20.3-57.7) |  | 0.729(0.289-1.840) | 0.503 |
| **rs1130409** |  |  | 0.346 |  |  |  |  | 0.222 |  |  |  |  | 0.082 |  |  |
| TT/TG | 27(87.1) | 18.0(6.2-29.8) |  | 1 |  |  | 33(14.1-51.9) |  | 1 |  |  | 31(14.8-47.2) |  | 1 |  |
| GG | 4(12.9) | ---- |  | 0.504(0.116-2.183) | 0.360 |  | -- |  | 0.306(0.040-2.310) | 0.251 |  | --- |  | 0.210(0.028-1.576) | 0.129 |
| **rs1805414** |  |  | **0.109** |  |  |  |  | 0.378 |  |  |  |  | 0.067 |  |  |
| AA/AG | 26(83.9) | 25(7.8-42.2) |  | 1 |  |  | 49(23.9-74.1) |  | 1 |  |  | 39(13.2-64.8) |  | 1 |  |
| GG | 5(16.1) | 8.0(5.9-10.1) |  | 2.402(0.790-7.310) | 0.123 |  | 16(11.7-20.3) |  | 2.447(0.782-7.655) | 0.124 |  | 16(11.7-20.3) |  | 2.522(0.901-7.061) | 0.078 |
| **rs2228001** |  |  | 0.452 |  |  |  |  | **0.027** |  |  |  |  | 0.019 |  |  |
| GG/GT | 20(64.5) | 18(5.7-30.3) |  | 1 |  |  | 26(15.6-36.4) |  | 1 |  |  | 23(9.8-36.1) |  | 1 |  |
| TT | 11(35.5) | 31(15.0-47.0) |  | 0.715(0.294-1.734) | 0.458 |  | -- |  | 0.292(0.092-0.927) | 0.037 |  | 51(48.1-53.9) |  | 0.338(0.128-0.890) | 0.028 |
| **rs873601** |  |  | 0.789 |  |  |  |  | 0.251 |  |  |  |  | 0.674 |  |  |
| GG/GA | 9(29.0) | 15(0-39.9) |  | 1 |  |  | -- |  | 1 |  |  | 50(0-111.4) |  | 1 |  |
| AA | 22(71.0) | 18(9.3-26.7) |  | 1.146(0.417-3.149) | 0.791 |  | 33(17.1-48.9) |  | 1.075(0.503-2.300) | 0.851 |  | 31(19.9-42.0) |  | 1.218(0.473-3.137) | 0.683 |
| **Co-dominant model** | |  |  |  |  |  |  |  |  |  |  |  |  |  |  |
| **rs1047768** |  |  | 0.951 |  |  |  |  | 0.638 |  |  |  |  | 0.656 |  |  |
| TT | 2(6.5) | --- |  | 1 |  |  | 39(16.4-61.6) |  | 1 |  |  | -- |  | 1 |  |
| TC | 15(48.4) | 15(7.7-22.3) |  | 0.793(0.170-3.711) | 0.769 |  | -- |  | 0.496(0.102-2.421) | 0.386 |  | 33(5.9-60.1) |  | 0.639(0.138-2.967) | 0.567 |
| CC | 12(38.7) | 22(1.5-42.5) |  | 0.869(0.183-4.122) | 0.860 |  | -- |  | 0.485(0.096-2.461) | 0.383 |  | 37(20.3-57.7) |  | 0.493(0.100-2.440) | 0.386 |
| **rs1130409** |  |  | 0.631 |  |  |  |  | 0.473 |  |  |  |  | 0.175 |  |  |
| TT | 12(38.7) | 22(10.5-33.5) |  | 1 |  |  | 33(23.7-42.3) |  | 1 |  |  | 31(14.0-47.9)) |  | 1 |  |
| TG | 15(48.4) | 16(0.8-31.1) |  | 1.085(0.436-2.703) | 0.861 |  | 25(0-56.3) |  | 1.024(0.378-2.773) | 0.963 |  | 25(0-56.3) |  | 0.732(0.298-1.798) | 0.496 |
| GG | 4(12.9) | -- |  | 0.528(0.111-2.507) | 0.422 |  | --) |  | 0.310(0.038-2.527) | 0.274 |  | -- |  | 0.174(0.022-1.397) | 0.100 |
| **rs1805414** |  |  | 0.259 |  |  |  |  | 0.279 |  |  |  |  | 0.169 |  |  |
| AA | 14(45.2) | 29(18.8-39.2) |  | 1 |  |  | 49(27.9-70.1) |  | 1 |  |  | 49(37.9-70.1) |  | 1 |  |
| AG | 12(38.7) | 16(11.5-20.5) |  | 1.204(0.454-3.193) | 0.709 |  | -- |  | 1.040(0.399-3.189) | 0.945 |  | 19(8.8-29.2) |  | 1.255(0.475-3.314) | 0.646 |
| GG | 5(16.1) | 8(5.6-10.1) |  | 2.588(0.791-8.470) | 0.116 |  | 16(11.7-20.3) |  | 2.484(0.733-8.415) | 0.144 |  | 16(11.7-20.3) |  | 2.772(0.909-8.451) | 0.073 |
| **rs2228001** |  |  | 0.598 |  |  |  |  | 0.034 |  |  |  |  | 0.039 |  |  |
| GG | 6(19.4) | 7(3.799-10.201) |  | 1 |  |  | 12(9.6-14.4) |  | 1 |  |  | 12(9.6-14.4) |  | 1 |  |
| GT | 14(45.2) | 22(6.106-37.894) |  | 0.684(0.209-2.242) | 0.531 |  | 26(15.8-36.2) |  | 0.500(0.150-1.665) | 0.259 |  | 25(19.5-30.5) |  | 0.592(0.184-1.905) | 0.379 |
| TT | 11(35.5) | 31(15.006-46.994) |  | 0.542(0.162-1.815) | 0.321 |  | -- |  | 0.170(0.039-0.729) | 0.017 |  | 51(48.1-53.9) |  | 0.219(0.057-0.842) | 0.027 |
| **rs873601** |  |  | 0.789 |  |  |  |  | 0.251 |  |  |  |  | 0.850 |  |  |
| GG | -- | -- |  | 1 |  |  | -- |  | 1 |  |  | -- |  | 1 |  |
| GA | 9(29.0) | 15(0-39.9) |  | 0.872(0.318-2.396) | 0.791 |  | -- |  | 0.489(0.140-1.710) | 0.263 |  | 50(0-111.4) |  | 0.821(0.319-2.114) | 0.683 |
| AA | 22(71.0) | 18(9.3-26.6) |  | -- | -- |  | 33(17.1-48.8) |  | -- | - |  | 31(19.9-42.0) |  | -- | -- |
| **Dominant model** | |  |  |  |  |  |  |  |  |  |  |  |  |  |  |
| **rs1047768** |  |  | 0.799 |  |  |  |  | 0.344 |  |  |  |  | 0.256 |  |  |
| TT | 2(6.5) | -- |  | 1 |  |  | -- |  | 1 |  |  | -- |  | 1 |  |
| TC/CC | 27(87.1) | 18(1.513-34.487) |  | 0.828(0.190-3.615) | 0.801 |  | 39(15.3-62.7) |  | 0.491(0.109-2.217) | 0.355 |  | 39(17.1-60.9) |  | 0.573(0.129-2.541) | 0.463 |
| **rs1130409** |  |  | 0.881 |  |  |  |  | 0.708 |  |  |  |  | 0.225 |  |  |
| TT | 12(38.7) | 22(10.5-33.5) |  | 1 |  |  | 33(23.7-42.3) |  | 1 |  |  | 31(14.0-47.9) |  | 1 |  |
| GT/GG | 19(61.3) | 16(1.8-30.2) |  | 0.935(0.387-2.261) | 0.882 |  | 49(9.9-88.1) |  | 0.831(0.314-2.200) | 0.709 |  | 49(9.9-88.1) |  | 0.583(0.241-1.412) | 0.232 |
| **rs1805414** |  |  | 0.361 |  |  |  |  | 0.490 |  |  |  |  | 0.264 |  |  |
| AA | 14(45.2) | 29(18.8-39.2) |  | 1 |  |  | 49(27.9-70.1) |  | 1 |  |  | 49(27.9-70.1) |  | 1 |  |
| AG/GG | 17(54.8) | 13(9.1-16.9) |  | 1.489(0.625-3.548) | 0.369 |  | 23(5.6-40.4) |  | 1.396(0.536-3.637) | 0.494 |  | 19(9.6-28.4) |  | 1.605(0.680-3.793) | 0.280 |
| **rs2228001** |  |  | 0.367 |  |  |  |  | 0.048 |  |  |  |  | 0.105 |  |  |
| GG | 6(19.4) | 7(3.8-10.2) |  | 1 |  |  | 12(9.6-14.4) |  | 1 |  |  | 12(9.6-14.4) |  | 1 |  |
| GT/TT | 25(80.6) | 22(8.3-35.7) |  | 0.609(0.203-1.825) | 0.376 |  | 39(13.1-64.9) |  | 0.330(0.104-1.054) | 0.061 |  | 39(16.6-61.3) |  | 0.403(0.129-1.259) | 0.118 |
| **rs873601** |  |  | n.a |  |  |  |  | n.a |  |  |  |  | -- |  |  |
| GG | 0(0) | - |  | 1 |  |  | -- |  | 1 |  |  | -- |  | 1 |  |
| GA/AA | 31(100) | 18(7.8-28.2) |  | -- | -- |  | 39(16.9-61.1) |  | -- | -- |  | 33(16.6-49.4) |  | -- | -- |

*，Kaplan–Meier survival analysis；#，univariate Cox regression analyses
